# Butein Inhibits Cell Growth by Blocking the IL-6/IL-6Rα Interaction in Human Ovarian Cancer and by Regulation of the IL-6/STAT3/FoxO3a Pathway

**DOI:** 10.3390/ijms24076038

**Published:** 2023-03-23

**Authors:** Sun-Ae Park, Young Ju Seo, Lee Kyung Kim, Hee Jung Kim, Kee Dong Yoon, Tae-Hwe Heo

**Affiliations:** 1 Laboratory of Pharmacoimmunology, Integrated Research Institute of Pharmaceutical Sciences and BK21 FOUR Team for Advanced Program for Smart Pharma Leaders, College of Pharmacy, The Catholic University of Korea, 43 Jibong-ro, Bucheon 14662, Republic of Korea; 2 College of Pharmacy, The Catholic University of Korea, Bucheon 14662, Republic of Korea

**Keywords:** ovarian cancer (OC), signaling pathway, interleukin-6 (IL-6), signal transducer and activator of transcription 3 (STAT3), forkhead box O3a (FoxO3a)

## Abstract

*Butea monosperma* (Fabaceae) has been used in traditional Indian medicine to treat a variety of ailments, including abdominal tumors. We aimed to investigate the anti-IL-6 activity of butein in ovarian cancer and elucidate the underlying molecular mechanisms. Butein was isolated and identified from *B. monosperma* flowers, and the inhibition of IL-6 signaling was investigated using the HEK-Blue™ IL-6 cell line. The surface plasmon resonance assay was used to estimate the binding of butein to IL-6, IL-6Rα, and gp130. After treatment with butein, ovarian cancer cell migration, apoptosis, and tumor growth inhibition were evaluated in vitro and in vivo. Furthermore, we used STAT3 siRNA to identify the mechanistic effects of butein on the IL-6/STAT3/FoxO3a pathway. Butein suppressed downstream signal transduction through higher binding affinity to IL-6. In ovarian cancer, butein inhibited cell proliferation, migration, and invasion, and induced cell cycle arrest and apoptosis. In addition, it decreased the growth of ovarian cancer cells in xenograft tumor models. Butein inhibited STAT3 phosphorylation and induced FoxO3a accumulation in the nucleus by inhibiting IL-6 signaling. The anticancer activity of butein was mediated by blocking the IL-6/IL-6Rα interaction and suppressing IL-6 bioactivity via interfering with the IL-6/STAT3/FoxO3a pathway.

## 1. Introduction

Ovarian cancer is the eighth most common cancer in women worldwide and ranks fifth in cancer death among women in the United States. Standard treatment consists of cell reduction surgery and platinum-based chemotherapy. However, it is a malignant cancer with high anticancer drug resistance and recurrence rate, so the 5-year survival rate is only 10–40% [1,2]. Therefore, novel therapeutic agents are required to reduce the chemotherapy resistance and recurrence of ovarian cancer.

*Butea monosperma* (Fabaceae) is a medium-sized deciduous tree which grows to about 50 feet and is native to Southeastern Asian nations such as Bangladesh, India, Thailand, and Western Indonesia [3,4,5]. The flowers of *B. monosperma* have traditionally been used as an astringent, diuretic, depurative, aphrodisiac, and tonic [6], and a variety of flavonoids (e.g., butein, butin, coreopsin, isobutrin, and monospermoside) have been identified from this plant. Previous studies have demonstrated that butein exerts anticancer activity and inhibits the proliferation of many human cancers, including colon, breast, hepatocellular, and cervical cancers [7,8,9]. Butein is involved in cell survival, proliferation, migration, invasion, and angiogenesis, and targets many molecular pathways in various cancers. Butein has also been shown to have immunomodulatory activity by inhibiting the expression of inflammatory mediators such as IL-6 and TNF-α in HaCaT cells, a keratinocyte cell line. It most commonly affects the expression of NF-κB and its downstream regulators [10,11]. Other important molecular targets include VEGF, STAT3, ERK, JNK, Akt, and p38 [9,12,13,14]. However, the role of butein in ovarian cancer has not been actively studied; in particular, there have been no studies that clearly explain its molecular mechanism. The fact that it is available as a treatment for ovarian cancer requires better insight into its pharmacological mechanisms. 

Interleukin-6 (IL-6) has been shown to have a direct stimulatory effect on many cancer cells through its action on several cell cycle pathways. IL-6 binds to the non-signaling IL-6 receptor (IL-6R) and then forms a complex with the signaling co-receptor glycoprotein 130 (gp130). IL-6 induced Janus Kinase (JAK)/signal transducer and activator of transcription 3 (STAT3) activation leads to constitutive activation of STAT3, which correlates with enhanced tumor cell growth and chemotherapy resistance [15,16,17]. This makes the IL-6/IL-6Rα/gp130 signaling pathway an attractive target for therapeutic or preventive intervention. The application of IL-6 blockers as anti-cancer agents has been investigated in many cancer types, but the only currently approved monoclonal antibodies (mAbs) in the United States are tocilizumab (anti-IL-6Rα) and siltuximab (anti-IL-6). These are intended for the treatment of rheumatoid arthritis (RA) and Castleman’s disease, not for use as cancer drugs. Currently, the application of IL-6/IL-6Rα/gp130 blockers as anti-cancer agents has not been extensively studied, much less for ovarian cancer. As disease progression depends on various IL-6-related mechanisms in ovarian cancer, the IL-6 signaling pathway is an ideal target for drug development. It was recently demonstrated that STAT3 regulates the expression of forkhead box class O 3a (FoxO3a) and the cell cycle regulatory proteins p27^kip1^ and p21^waf1^ [18,19]. FoxO3a is a transcription factor that mediates several physiological and pathological processes by regulating gene expression in apoptosis, proliferation, cell cycle progression, and DNA damage [20]. Therefore, further studies on the effect of IL-6 blockers in ovarian cancer are needed and elucidating the correlation between IL-6 activated STAT3 phosphorylation and FoxO3a may suggest and exciting new therapeutic directions. 

In this study, we investigated the therapeutic potential of butein isolated from *B. monosperma* flowers in ovarian cancer. We aimed to elucidate the anti-IL-6 property of butein, which blocks the interaction between IL-6 and IL-6Rα, and to elucidate the underlying molecular mechanisms involved in the regulation of the STAT3 pathway. 

## 2. Results

### 2.1. Identification of Compounds ***1**–**14*** from Butea monosperma Flowers

**Figure 1:**
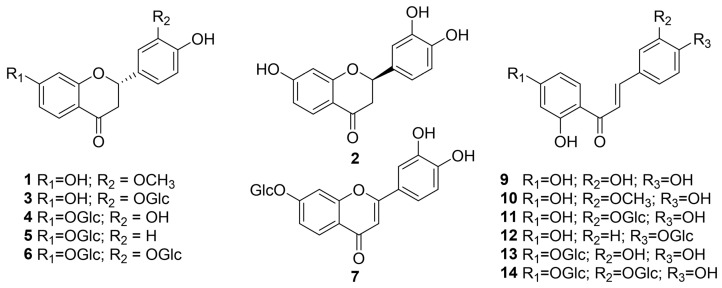
Chemical structures of compounds **1**–**14** isolated from *Butea monosperma* flowers.

The CH_2_Cl_2_- and EtOAc-soluble extract extracts of *B. monosperma* flowers were subjected to multi-step column chromatography to yield 14 known compounds. The chemical structures of these isolates were identified by spectroscopic evidence to be liquiritigenim-5′-*O*-methyl ether (1) [21], (+)-butin (2) [22], (−)-isomonospermoside (3) [23], isocoreopsin (4) [23], liquiritigenin 7-*O*-beta-D-glucopyranoside (5) [24], (−)-butrin (6) [25], 3′, 4′, 7-trihydroxyflavone-7-*O*-glucoside (7) [26], isoliquilitigenin (8) [27], butein (9) [28], homobutein (10) [29], monospermoside (11) [23], isoliquilitin (12) [28], coreopsin (13) [30], and isobutrin (14) [23] (Figure 1 and Supplementary Information (SI)).

### 2.2. Characterization of Butein and Anti-IL-6 Activity In Vitro

**Figure 2:**
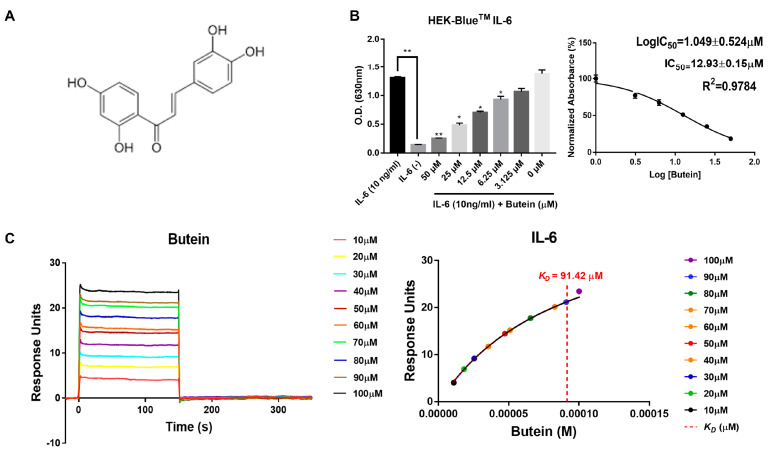
Characterization of butein and its anti-IL-6 activity in vitro. (**A**) Chemical structure of butein. (**B**) HEK-Blue™ IL-6 cells were treated with various concentration of butein for 24 h. The activation of IL-6 was measured by a SEAP activity assay after the treatment of HEK-Blue™ IL-6 cells with the different indicated concentrations of butein for 1 h in the presence or absence of IL-6 for 24 h (* *p* < 0.05, ** *p* < 0.01). (**C**) For SPR analysis, IL-6 was immobilized on a CM5 sensor chip, and butein was injected into the flow cells. T-200 BIAevaluation software (v3.0) was used to subtract the references and determine the steady-state KD. IL-6, interleukin 6.

The HEK-Blue™ IL-6 cell line was used to find isolates having an inhibitory effect on IL-6 signaling among the 14 isolates from *B. monosperma*. The cells were treated with increasing concentrations of the 14 isolates (0, 3.125, 6.25, 12.5, 25, and 50 μM) in the presence of 10 ng/mL IL-6. After 24 h of reaction, the secreted embryonic alkaline phosphatase (SEAP) signal of HEK-Blue™ IL-6 was inhibited in a concentration-dependent manner by the isolates (Figure S1), with butein (compound **9**) achieving the maximum inhibition (Figure 2B). The HEK- Blue™ IL-6 cell bioassay showed that butein blocked IL-6 induced bioactivity. We found that butein interfered with the interaction between IL-6 and IL-6Rα and reduced it by 21.7% at 20 μM (Figure S2A). We then performed SPR to confirm if butein could directly bind to IL-6. After each IL-6, IL-6Rα, and gp130 protein was immobilized on the CM5 chip, the binding affinity between the immobilized protein and butein was examined. We found that butein had binding affinity to immobilized IL-6 protein (*K_D_* = 91.42 μM; Figure 1C). In addition, to investigate the association between IL-6Rα and gp130 binding affinity, the RU for each concentration of butein was detected through SPR analysis, and the results were added to Figure S2B. We also compared the binding behaviors of butein to IL-6Rα with that to gp130 protein. Butein showed binding affinity to im-mobilized IL-6Rα and gp130 proteins (*K_D_* = 435.3 μM and *K_D_* = 615.7 μM, respectively; Figure S2B). It associates faster with IL-6 than with IL-6Rα and gp130, thus indicating that it has a lower affinity to IL-6Rα and gp130 protein. As a result, butein inhibits downstream signaling through higher binding affinity to IL-6.

### 2.3. Butein Suppresses the Cell Viability, Migration, and Invasion of Ovarian Cancer Cells

**Figure 3:**
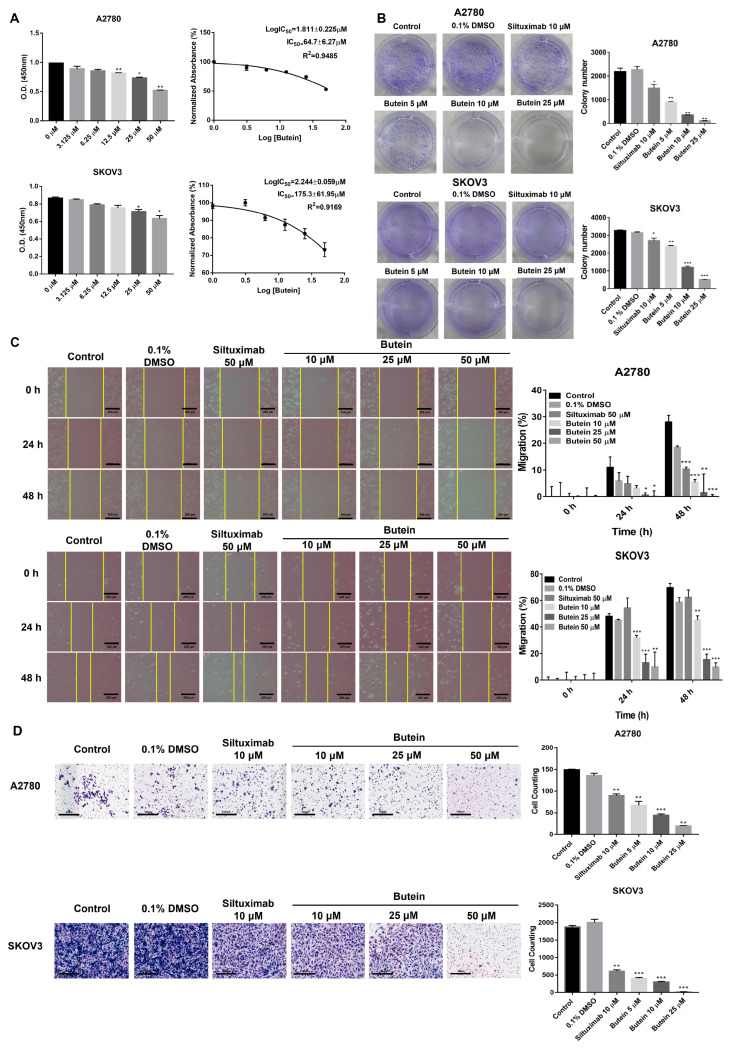
Butein inhibited cell viability, proliferation, migration, and invasion of ovarian cancer cells. (**A**) A2780 and SKOV3 cells were treated with butein at the indicated concentrations in triplicate for 48 h and processed for MTT assay to analyze cell viability (* *p* < 0.05, ** *p* < 0.01). (**B**) Clonogenic assay revealed that butein dramatically reduced the colony growth of ovarian cancer cells (* *p* < 0.05, ** *p* < 0.01, *** *p* < 0.001). (**C**) A2780 and SKOV3 cells were treated with butein and allowed to migrate to the scratched area for 24 h and 48 h. Yellow color lines indicate a gap in the scratched area. The percentage of migrating area in the wound-healing assay quantified in A2780 and SKOV3 cells is shown (* *p* < 0.05, ** *p* < 0.01, and *** *p* < 0.001). (**D**) Matrigel invasion assay was used to determine the invasion of A2780 and SKOV3 cells after 48 h of butein treatment. Bars indicate mean ± standard deviation of three independent experiments performed in triplicate (** *p* < 0.01 and *** *p* < 0.001).

To investigate the effects of butein on ovarian cancer in vitro, A2780 and SKOV3 cell lines were used. Cell viability of A2780 and SKOV3 cells was inhibited with IC50 values of 64.7 ± 6.27 μM and 175.3 ± 61.95 μM, respectively, upon butein treatment (Figure 3A). Similarly, their clonogenicity was significantly inhibited in the butein-treated cells compared with the control siltuximab (anti-IL-6)-treated cells (Figure 3B). The wound healing assay indicated that butein reduced cell migration at 24 h and 48 h in a concentration-dependent manner compared with the control, 0.1% DMSO-, and siltuximab-treated group (Figure 3C). Cell infiltration was similarly affected as observed in the Matrigel cell invasion assay (Figure 3D).

### 2.4. Butein Induced Ovarian Cancer Cell Cycle Arrest and Cell Apoptosis

**Figure 4:**
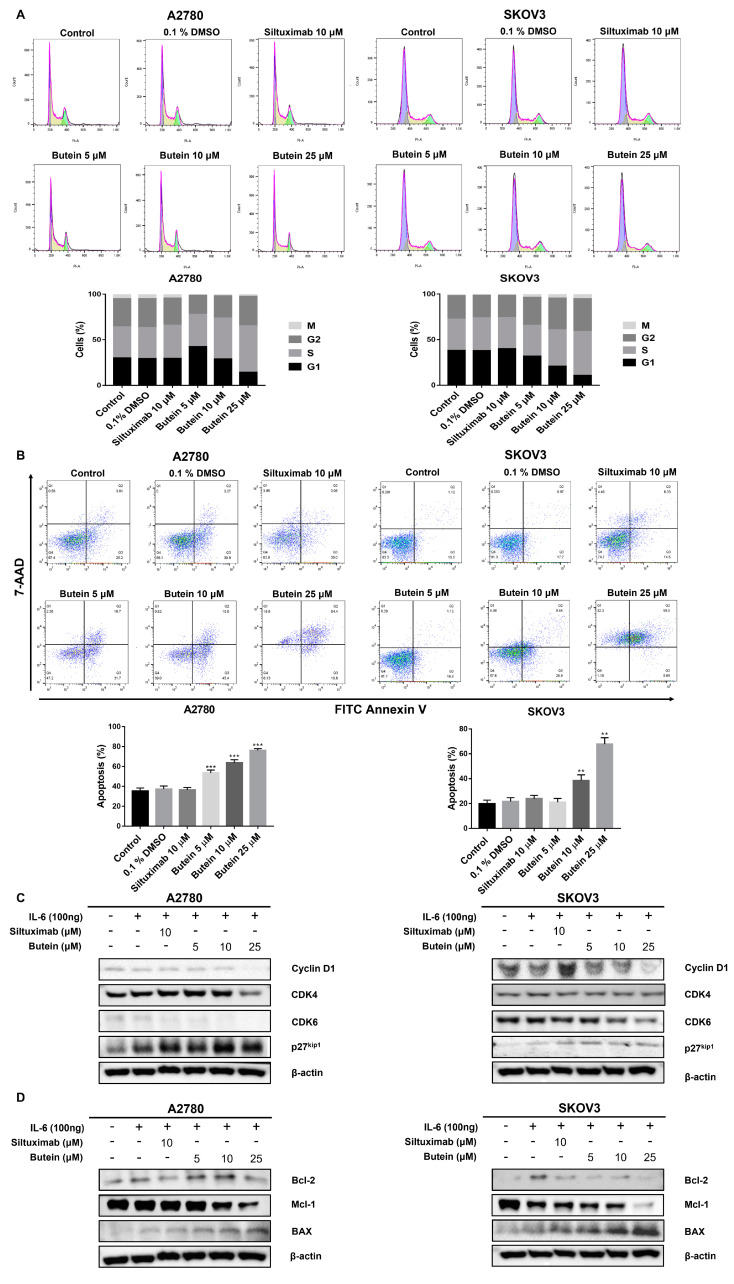
Butein induced growth arrest and apoptosis of ovarian cancer cells. (**A**) Butein induced cycle arrest in ovarian cancer cells, which were stained by PI, and cell cycle distribution was analyzed by flow cytometry. (**B**) A2780 and SKOV3 cells were treated with various concentrations of butein in triplicate for 48 h to detect cell apoptosis using the FITC Annexin V apoptosis Kit (** *p* < 0.01 and *** *p* < 0.001). (**C**) Butein decreased the expression of Cyclin D1, CDK 4, CDK 6, and increased the expression of p27^kip1^ in A2780 and SKOV3 cells. (**D**) Butein decreased the expression of Bcl-2, Mcl-1, and increased the expression of Bax in A2780 and SKOV3 cells.

Cell cycle progression was monitored using flow cytometry. Exposure to butein resulted in an increase in G1-phase cells along with a decrease in S-phase cells. The effect was observed with 25 μM butein treatment, leading to 55.9% of A2780 cells in G1-phase vs. 45.3% under control conditions. Similarly, 65.8% of SKOV3 cells upon butein treatment and 58.3% under control conditions were in G1-phase (Figure 4A). Butein was also found to significantly increase total apoptosis in both A2780 and SKOV3 cell lines in a dose-dependent manner (*p* < 0.005) (Figure 4B). Consequently, our results demonstrated that treatment with butein inhibits the growth of ovarian cancer cells by inducing cell cycle arrest and apoptosis.

In accordance with the above data, Western blot analysis indicated that the expression levels of the cell cycle proteins CDK4, CDK6, and Cyclin D1 were reduced, while that of p27kip1 was enhanced, in the butein-treated A2780 and SKOV3 cell lines in a dose-dependent manner (Figure 4C). Meanwhile, the pro-apoptosis protein Bax was upregulated while Bcl-2 and Mcl-1 were downregulated in the butein-treated A2780 and SKOV3 cell lines in a dose-dependent manner (Figure 4D). Whole membrane and protein expression levels detected by Western blotting are shown in Figure S3. These data suggest that butein regulates cell fate by modulating the expression of cell cycle and apoptosis proteins.

### 2.5. Butein Inhibited STAT3 Phosphorylation and Induced Intranuclear Accumulation of FoxO3a through Inhibition of IL-6 Signaling

**Figure 5:**
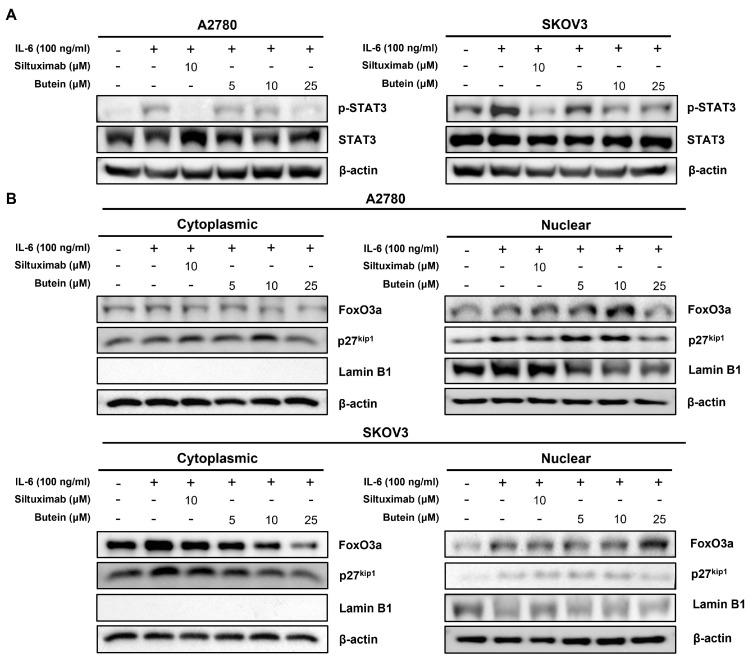
Butein inhibited STAT3 phosphorylation and induced intranuclear accumulation of FoxO3 through inhibition of IL-6 signaling. (**A**) The phosphorylation levels of STAT3 were decreased in the butein-treated group. (**B**) The upregulated levels of FoxO3a and p27^kip1^ in the nucleus and the downregulated levels of p27^kip1^ in the cytoplasm were determined by Western blotting of A2780 and SKOV3 cell extracts. β-actin served as the loading control.

To further elucidate the effect of IL-6 inhibition by butein, the expression of the downstream gene, STAT3, was analyzed by Western blotting of proteins from the A2780 and SKOV3 cell lines after treatment with varying concentrations of butein. No significant change was observed in the total amount of STAT3. However, the phosphorylation of STAT3 was found to be inhibited in a concentration-dependent manner in the butein-treated cells relative to the IL-6 treated cells (Figure 5A). Whole membrane and protein expression levels detected by Western blotting are shown in Figure S4. Western blot analysis of the nuclear and cytoplasmic protein extracts revealed that butein also induced FoxO3a accumulation in the nucleus and decreased cytoplasmic FoxO3a. Consistent with this, expression of the proliferation-related gene p27^kip1^ was also found to be enhanced in the nucleus (Figure 5B). Whole membrane and protein expression levels detected by Western blotting are shown in Figure S5. These results indicate that butein induces STAT3 inactivation through the inhibition of IL-6 signaling in ovarian cancer and increases FoxO3a and p27^kip1^ levels in the nucleus following STAT3 inactivation.

### 2.6. Butein Increased Protein Expression of FoxO3a and p27^kip1^ through Inactivation of STAT3

**Figure 6:**
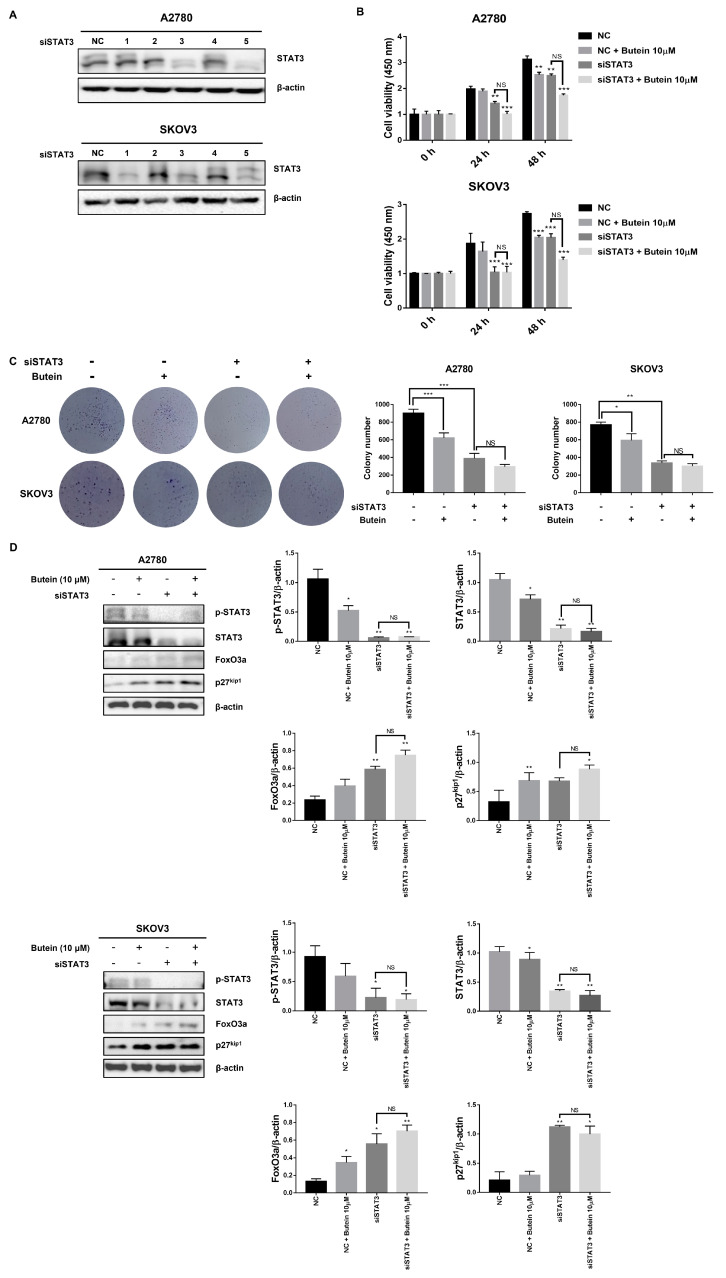
Butein increased protein expression of FoxO3a and p27^kip1^ through inactivation of STAT3. (**A**) Western blot showing STAT3 protein expression following siSTAT3 treatment. β-actin was used as a loading control. (**B**) Cell proliferation upon butein and siSTAT3 treatment was detected through the CCK-8 assay (** *p* < 0.01 and *** *p* < 0.001; NS, not significant). (**C**) Cell growth following butein and siSTAT3 treatment was detected by colony formation assay (* *p* < 0.05, ** *p* < 0.01, and *** *p* < 0.001; NS, not significant). Data represent the mean ± SD of three replicates. (**D**) Expression levels of STAT3, FoxO3a, and p27^kip1^ proteins in ovarian cancer cells with the combination of butein and siSTAT3 treatment. β-actin was used as a loading control.

To elucidate the mechanism of butein’s effect on FoxO3a and p27^kip1^, we used siRNA-mediated knock-down of STAT3. Based on the Western blot results, siSTAT3-5—which had the highest knock-down efficiency among the siSTAT3 primers—was selected (Figure 6A). When the ovarian cancer cell line was treated with siSTAT3, the cell growth inhibition was similar to that in the butein-treated group. The inhibitory effect was enhanced when butein and siSTAT3 were administered together (Figure 6B). Treatment of butein and siSTAT3 collectively in ovarian cancer cell lines inhibited colony formation, while no significant difference was found between groups treated separately with siSTAT3 and butein (Figure 6C). These data demonstrate that butein exerts antiproliferative effects on ovarian cancer cell lines through STAT3. Additionally, FoxO3a and p27^kip1^ protein levels were found to be upregulated upon STAT3 knock-down, as in the butein-treated group (Figure 6D). Whole membrane and protein expression levels detected by Western blotting are shown in Figure S6. These data show that butein treatment not only mimics the downstream effects of STAT3 suppression but also enhances them, suggesting that butein affects FoxO3a and p27^kip1^ through STAT3 inactivation.

### 2.7. Butein Exerts an Antitumor Effect In Vivo on Ovarian Cancer Cells

**Figure 7:**
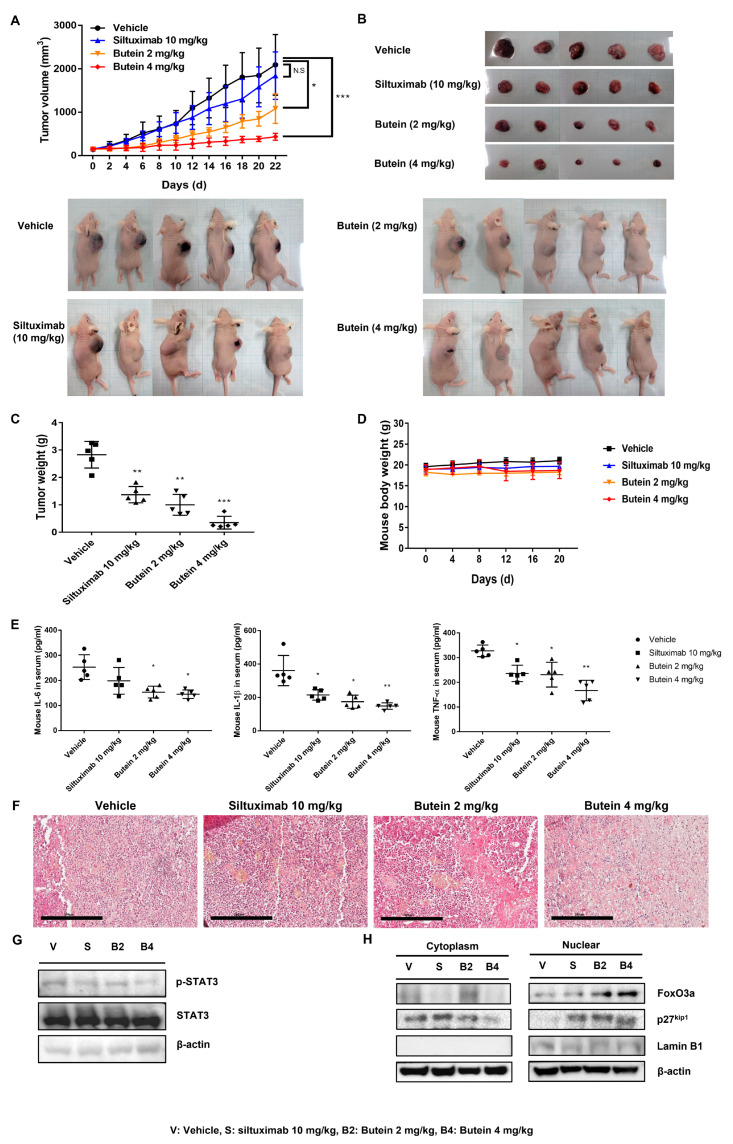
Butein inhibited A2780 and tumor growth in vivo. A2780 cells (1 × 10^7^) were injected subcutaneously into BALB/c nude mice with an equal volume of PBS. When tumors reached a volume of 150 mm^3^, siltuximab (10 mg/kg) or butein (2 or 4 mg/kg) in a vehicle of 0.05% CMC and 5% DMSO was administered by intraperitoneal injection five times a week. (**A**) Tumor volumes were calculated from caliper measurements (* *p* < 0.05, *** *p* < 0.001). (**B**) The tumor masses were excised for comparison between the groups. (**C**) After 21 days of treatment, all mice were sacrificed, and the total mass of each tumor was determined at autopsy (n = 5 mice per treatment group) (** *p* < 0.01, *** *p* < 0.001). (**D**) The mouse body weight measured on the days indicated. (**E**) To assess IL-6, IL-1β, and TNF-α production in serum in mouse blood, the total amount of IL-6, IL-1β, and TNF-α were normalized to the vehicle (* *p* < 0.05, ** *p* < 0.01). (**F**) H&E staining results show the anticancer effect of butein on ovarian cancer. (**G**) The phosphorylation levels of STAT3 were determined using Western blotting of the harvested tumor tissue. β-actin served as a loading control. (**H**) The upregulated levels of FoxO3a and p27^kip1^ were determined using Western blotting of nuclear extracts of the harvested tumor tissue. β-actin and Lamin B1 served as loading controls.

To test the effect of butein on tumor growth inhibition in vivo, we generated xenograft mice using the A2780 cell line. The butein-treated group showed a significant inhibition of tumor growth compared with the vehicle as well as the siltuximab-treated group (Figure 7A). The tumor mass also showed a similar trend (Figure 7B,C). There was no difference in the total mouse weight during the experimental period (Figure 7D). To evaluate the production of IL-6, IL-1β, and TNF-α in serum in mouse blood, the total amount of IL-6, IL-1β, and TNF-α was normalized to the total amount of vehicle (Figure 7E). In the vehicle group, tumor cells were closely arranged into complete and atypical structures. In contrast, the group treated with a high concentration of butein displayed a greater degree of tumor cell death, characterized by an incomplete cell membrane, a pyknotic nucleus, and condensed cytoplasm. Supporting the in vitro experimental results, we identified similar changes in protein expression upon butein treatment in vivo (Figure 7G,H). Butein-mediated inhibition of STAT3 phosphorylation as well as increased nuclear FoxO3a were observed in mouse tumor tissues. Whole membrane and protein expression levels detected by Western blotting are shown in Figure S7. These data suggested that butein treatment inhibited tumor growth in ovarian cancer cells by increasing the nuclear accumulation of FoxO3a through the inhibition of STAT3 phosphorylation in vivo.

## 3. Discussion

Ovarian cancer is the most lethal gynecological malignancy, and inflammation has been shown to play a large role in ovarian cancer growth. IL-6 is a cytokine that acts on chronic inflammation as a major tumor-promoting inflammatory mediator. IL-6 has been shown to activate signaling pathways leading to tumor proliferation, the most studied of which are the JAK and STAT3 pathways. Many drugs were found to inhibit IL-6 signaling, including siltuximab and sirukumab [31], although, none of them currently show promising outcomes in ovarian cancer treatment. Therefore, we aimed to discover new small molecule inhibitors of IL-6 signaling and to elucidate their mechanisms of action. 

The 14 compounds isolated and identified from *Butea monosperma* flowers are in accordance with previous studies [21,22,23,24,25,26,27,28,29,30]. Of these, butein shows anti-inflammatory activity and has been shown to be a potential therapeutic agent for the treatment of chronic inflammatory diseases and cancers [12]. Recent evidence suggests that it inhibits the activities of anti-inflammatory cytokines such as IL-6, IL-1β, and TNF-α [13]. However, whether it directly inhibits anti-inflammatory cytokines has not been studied. Using the SPR assay, we confirmed that butein binds to IL-6, IL-6Rα, and gp130 through intermolecular interactions. We further validated that the binding force between butein and IL-6 was higher, and that butein inhibited IL-6 downstream signaling using the HEK-Blue™ IL-6 cell line. Ours is the first study to report that butein binds to IL-6 and inhibits its downstream signaling. Furthermore, our data indicating the low expression of these cytokines in the mouse blood serum following butein treatment supports these previous findings. 

Our results showed that survival, migration, and invasion of ovarian cancer cells were inhibited by butein, and that this occurred in a concentration-dependent manner. Furthermore, treatment with increasing concentrations of butein resulted in increased cell cycle arrest as well as increased apoptosis. Further investigation revealed that butein affects the expression levels of cell cycle proteins as well as apoptosis proteins. Along similar lines, previous studies have shown that butein inhibits the activation of various oncogenes through many signaling mechanisms [12,32]. A recent report revealed that butein can exert a chemosensitizing effect through the miR-186-5p-TWIST1 axis, suggesting that butein exerts its chemosensitizing effect, at least in part, through microRNA modulation [33].

Butein is known to exhibit anticancer effects by the inhibition of STAT3, Akt, and PI3K signaling in other cancers as well [9,13,34]. Our in vitro as well as in vivo data suggests that the same molecular pathways are affected by butein via similar mechanisms in ovarian cancer. Additionally, it has been reported that, following IL-6 signaling, phosphorylated STAT3 regulates the nuclear translocation of FoxO3a, leading to increased expression of p27^kip1^ in T cells [19], similar to the mechanisms we found in ovarian cancer cells. Furthermore, recent evidence suggests that butein leads to increased p27^kip1^ expression by promoting not only the nuclear localization of FoxO3a, but also by enhancing its binding to the p27^kip1^ promoter, resulting in cell cycle arrest and thereby inhibiting cell proliferation [18,35]. We found that butein increased the expression of p27^kip1^ by regulating the mechanism of STAT3 and FoxO3a in ovarian cancer. According to a recent study, FoxO3a is an important regulator during the development of drug resistance and may show great potential as a novel biomarker for prognostic evaluation and therapeutic targets in cancer patients [36,37,38]. Additionally, FoxO3a exhibits great therapeutic potential due to its essential role in cancer progression, particularly in drug resistance. 

In summary, butein from *Butea monosperma* flower isolates show excellent therapeutic potential in ovarian cancer cells. It has shown anti-IL-6 activity, which induces inactivation of STAT3 through IL-6 binding and nuclear accumulation of FoxO3a in ovarian cancer cells. This suggests that butein, along with current front-line chemotherapy drugs, may represent a promising approach towards ovarian cancer treatment; however, this requires further research.

## 4. Materials and Methods

### 4.1. General Experimental Procedures

Preparative HPLC was performed on a Gilson HPLC system (Middleton, WI, USA) which consisted of a binary pump, a liquid handler, a UV/Vis detector and a Luna C18(2) column (21.2 × 250 mm I.D., 5 μm, phenomenex, Torrance, CA, USA). Silica gel 60 (40–63 μm, Merck, Germany) and ZEOprep 90 C18 (40–63 μm, Zeochme, Uetikon, Switzerland) were used for column chromatography. The semi-preparative scale high-performance countercurrent chromatography (HPCCC) instrument used was a Spectrum instrument (Dynamic Extractions, Berkshire, UK). The HPCCC system was composed of an IOTA 300 s pump (ECOM, Prague, Czech Republic), a Sapphire 600 UV–VIS detector (ECOM, Prague, Czech Republic) and a Foxy R2 fraction collector (Teledyne Isco, Lincoln, NE, USA). LC–MS data were obtained using an Agilent 6530 Q-TOF LC/MS system (Agilent Technologies, Santa Clara, CA, USA). One-dimensional (1D) and two-dimensional (2D) NMR data were recorded on an Avance 500 spectrometer (Bruker, Karlsruhe, Germany). Organic solvents were purchased from Dae-Jung Chemical Co., Ltd. (Seoul, Republic of Korea).

### 4.2. Plant Material

Flowers of *B. monosperma* were collected from Popa Mountain National Park in March 2014, and the specimen of *B. monosperma* was identified by Khin Myo Htwe (Popa Mountain National Park). A voucher specimen (#PopaButea_M 032013) of this plant was deposited at the Herbarium of the College of Pharmacy, the Catholic University of Korea.

### 4.3. Extraction and Isolation

The dried flowers of *B. monosperma* (157 g) were ground into a fine powder and extracted with methanol (2 L× 90 min × three times) to give a methanol-soluble extract (80.4 g). The methanol-soluble extract was suspended in H_2_O (1 L) and partitioned with organic solvents to produce CH_2_Cl_2_- (4.2 g), EtOAc- (4.3 g), and *n*-BuOH- (49.6 g) soluble extracts The CH_2_Cl_2_-soluble extract was subjected to silica gel medium-pressure column chromatography (MPLC) using a CH_2_Cl_2_–MeOH mixture (40:1 → 0:100 (*v*/*v*)) to give 16 sub-fractions (BMD1-BMD16). BMD8 (114.8 mg) was purified by RP-HPLC using a MeCN–H_2_O mixture (45:55 (*v*/*v*)) to yield **1** (21.8 mg). BMD10 (142.2 mg) was purified by RP-HPLC using 48:52 (*v*/*v*) of a MeCN–H_2_O mixture to produce **8** (35.2 mg). BMD13 (270.1 mg) was subjected to RP-MPLC using a gradient elution of a MeCN–H_2_O mixture (20:80 (*v*/*v*) → 90:10 (*v*/*v*)) to yield **9** (124.6 mg). BMD14 (424 mg) was subjected to RP–MPLC with a gradient elution of a MeCN–H_2_O mixture (10:90 (*v*/*v*) → 70:30 (*v*/*v*)) to yield **2** (145.6 mg). BMD6 (593.3 mg) was resolved into seven sub-fractions (BMD6-1 to BMD6-7) using RP-MPLC (MeCN–H_2_O, 10:90 (*v*/*v*) → 90:10 (*v*/*v*)). BMD6-4 (20.4 mg) was purified by RP-HPLC using a MeCN–H_2_O mixture (48:52 (*v*/*v*)) to produce **10** (7.3 mg). The EtOAc-soluble extract (4.27g) was subjected to silica gel-MPLC with a mixture of CH_2_Cl_2_–MeOH (5:1 *v*/*v*), resulting in four sub-fractions (BME1-BME4). BME3 was separated by RP-MPLC using a gradient elution of a MeCN–H_2_O mixture (15:85 (*v*/*v*) → 50:50 (*v*/*v*)) to obtain eight sub-fractions (BME3-1 to BME3-8). BME3-4 (39.3 mg) was purified by RP-HPLC (MeCN–H_2_O, 25:75 (*v*/*v*)) to yield **3** (19.7 mg). BME3-5 (106 mg) was subjected to RP-MPLC with 20:80 (*v*/*v*) of a MeCN–H_2_O mixture, resulting in three sub-fractions (BME3-5-1 to BME3-5-3). Compounds **11** (15.6 mg) and **12** (3 mg) were purified from BME3-5-3 using RP-HPLC with 35:65 (*v*/*v*) of a MeCN–H_2_O mixture. Compounds **7** (8.9 mg), **4** (16.6 mg) and **5** (0.5 mg) were separated by RP-HPLC using isocratic elution with a MeCN–H_2_O mixture (23:77 (*v*/*v*)). BME4 (32.8 mg) was resolved by RP-HPLC using a MeCN–H_2_O mixture to yield **13** (11.6 mg). The *n*-BuOH-soluble extract (200 mg) was subjected to high-performance column chromatography using a biphasic solvent system (EtOAC*n*–BuOH–H_2_O, 5:5:10 (*v*/*v*/*v*); rotation speed—1500 rpm; flow rate—3 mL/min; RP mode) to yield **6** (20.3 mg) and **14** (18.4 mg).

### 4.4. IL-6 Inhibitory Bioassay with HEK-Blue™ IL-6 Cells

HEK-Blue™ IL-6 cells were obtained from InvivoGen (San Diego, CA, USA). In a 96-well plate, 20 μL butein at various concentrations (0, 3.125, 6.25, 12.5, 25, and 50 μM) were added to the cell suspension (50,000 cells/200 μL). Following this, the recombinant human IL-6 (BioLegend, San Diego, CA, USA) was administered at 10 ng/mL. After 24 h, 20 μL of the IL-6-treated cell suspension was added to 180 μL of QUANTI-Blue™ Solution (JnvivoGen, San Diego, CA, USA) in a new 96-well plate. After 2 h of incubation at 37 °C, the spectrophotometric absorbance between 620 and 655 nm was recorded.

### 4.5. Surface Plasmon Resonance Assay

The surface plasmon resonance (SPR) assay was measured using the BIAcore T200 model (GE Healthcare, Chicago, IL, USA) with HBS-EP buffer (1×HBS-EP, GE Healthcare, Chicago, IL, USA) containing 5% dimethyl sulfoxide (DMSO; Sigma-Aldrich, St. Louis, MO, USA). The pH scouting for IL-6 (PeproTech, Rocky Hill, NJ, USA), IL-6Rα (PeproTech, Rocky Hill, NJ, USA), and gp130 (Sino Biological, Beijing, China, or ANRT, Daejeon, Korea) immobilization was performed in 10 mM acetate buffer at pH 4.5. IL-6, IL-6Rα, and gp130 were immobilized on a CM5 chip at 750, 1200, and 2000 response units (RU). Butein was injected into the IL-6, IL-6Rα and gp130-immobilized flow cell at concentrations of 10, 20, 30, 40, 50, 60, 70, 80, 90, and 100 μM with a 25 μL/min flow rate for 150 s and allowed to dissociate for 300 s. Steady-state KD was determined via the T-200 BIAevaluation software (GE Healthcare, Chicago, IL, USA).

### 4.6. Analysis of Cell Viability by the MTT Assay

A2780 and SKOV3 cell lines were purchased from the European Collection of Cell Cultures (ECACC, London, UK) and Korean Cell Line Bank (KCLB, Seoul, Republic of Korea) respectively. Each cell line was seeded in a 96-well plate at an appropriate cell number (3000 cells/100 μL in each well). After 24 h, butein was added to the plates at each of the following concentrations, 0, 3.125, 6.25, 12.5, 25, and 50 μM, in triplicate in the presence of serum medium. After 48 h, 100 μL of *N*, *N*-dimethylformamide (Sigma-Aldrich, St. Louis, MO, USA) solubilizing solution was added to each well and incubated for 4 h. The solution was then aspirated, and 100 μL of DMSO was added for cell lysis. The spectrophotometric absorbance was measured at 540 nm. Half of the maximum inhibitory concentration (IC_50_) was determined using GraphPad Prism software (version 7.0).

### 4.7. Clonogenic Formation Assay

A2780 and SKOV3 cell lines were seeded at approximately 5000 cells/well in 24-well plates. Butein was added to the cell medium at various concentrations (5, 10, and 25 μM) together with control siltuximab (CTNO328; EUSA Pharma, Inc., Hemel Hempstead, UK) for 12 h. After 2 weeks, the colonies formed were fixed with fixer buffer, stained with crystal violet, and imaged with a ChemiDoc imaging system (Bio-Rad, Herles, CA, USA).

### 4.8. Wound-Healing Assay

A2780 and SKOV3 cells were cultured to 90% confluence in 6-well plates. After changing to a serum-free medium, the cell monolayer was scraped to artificially form homogeneous wound with uniform thickness. The drug treatment for each well was carried out in the following manner: siltuximab at 50 μM; butein at 10, 25, and 50 μM. The cells were imaged at 0, 24, and 48 h.

### 4.9. Matrigel Invasion Assay

Matrigel invasion assays were performed using a BioCoat Matrigel Invasion Chamber (BD Bioscience, Bedford, MA, USA). A2780 and SKOV3 cells (50,000 cells) were placed inside the chamber in serum-free medium containing 500 μL of drugs, and 700 μL of medium containing serum were placed outside the chamber. After incubation for 48 h, the infiltrating cells were stained using the Differential Quick Stain Kit (Cat.no. 26096-50, Electron Microscopy Sciences, Hatfield, PA, USA). Cells were imaged with a Slide Scanner (Aperio CS2, Leica Microsystems, Wetzlar, Germany) and the number of invading cells was quantified using Image J.

### 4.10. Flow Cytometric Analysis of Cell Cycle and Apoptosis

A2780 and SKOV3 cell lines were treated with control (siltuximab 10 μM) and butein (5, 10, and 25 μM). After 6 h, the cells were fixed with 70% ethanol for cell cycle analysis and stained with propidium iodide (PI). Cell death was analyzed using the FITC Annexin V Apoptosis Detection kit with 7-AAD (Cat.no. 640922, BioLegend, San Diego, CA, USA). Cells were sorted using a FACS Canto II (BD Biosciences, Franklin Lakes, NJ, USA) and cell cycle and apoptosis were analyzed using BD FACS Diva software version 6. 2. Analysis (BD Biosciences, Franklin Lakes, NJ, USA) was performed in triplicate.

### 4.11. Small Interfering RNA (siRNA) Transfection

The siRNA siNC was used as a negative control, and siSTAT3 was used as a target siRNA. The siRNAs used were produced by Genolution (Genolution Pharmaceutical lnc., Seoul, Korea) and were transfected according to the manufacturer’s instructions. Target sequences for siRNAs are listed in Table S1.

### 4.12. Western Blotting

After each drug treatment of the cells, the cells were dissolved in 100 μL of radioimmunoprecipitation assay (RIPA) buffer (Thermo Fisher Scientific Inc., Waltham, MA, USA) and centrifuged to separate the supernatant containing protein. Isolated protein concentrations were measured using the Pierce BCA Protein Assay Kit (Thermo Fisher Scientific Inc., Waltham, MA, USA). Aliquots of proteins quantified at 30 ng were boiled at 100 °C in 5× sample buffer, separated on a 12% SDS-polyacrylamide gel and electrophoresed onto polyvinylidene difluoride (PDVF) membranes (Millipore, Billerica, MA, USA). The PDVF membranes were blocked for 1 h at 25 °C with 5% BSA in 1× tris buffered saline containing 0.05% Tween20 (TBST) (Sigma-Aldrich, St. Louis, MO, USA). The membrane was incubated in the primary antibody at 4 °C overnight. PDVF membranes were washed with 1× TBST and incubated with AffiniPure goat anti-rabbit IgG secondary antibody (Jackson Immunoresearch, West Grove, PA, USA) for 1 h at room temperature. After washing again with TBST buffer, enhanced signals were detected using the SuperSignal™ West Femto Maximum Sensitivity Substrate kit (cat.no. 34094, Thermo Scientific, Rockford, IL, USA). The blot was imaged using the ChemiDoc imaging system (Bio-Rad, Herles, CA, USA), and protein expression was quantified with the ImageJ software (v1.8.0).

The following primary antibodies were used: anti-β-actin (cat. No. 8457S; dilution 1:1000), anti-Bcl-2 (cat. no. 3498S; 1:500), anti-Bax (cat. no. 2772S; 1:1000), anti-Mcl-1 (cat. no. 4572S; 1:1000), anti-Cyclin D1 (cat. no. 2978T; 1:1000), anti-CDK4 (cat. no. 12790T; 1:1000), anti-CDK6 (cat. no. 3136T; 1:1000), anti-p27^kipl^ (cat. no. 3686T; 1:1000), anti-phospho-STAT3 (Tyr705) (cat. no. 9131S; 1:500), anti-STAT3 (cat.no. 12640S; 1:500), anti-FoxO3a (cat. no. 2497S; 1:1000), and Lamin B1 (cat.no. 12586S, 1:1000) (all from Cell Signaling Technologies, Danvers, MA, USA).

### 4.13. Mouse Xenografts

Adult BALB/c nude mice aged 5 weeks (n = 5, body weight 17−19 g) were purchased from Orient Bio (Seongnam, Republic of Korea) and reared in aseptic conditions with 55 ± 10% humidity and 25 ± 2 °C temperature under a 12 h/12 h light-dark cycle (Catholic University protocol). All animal experimental work was carried out in compliance with the legal obligations and federal guidelines and legal obligations for the care and maintenance of laboratory animals. All animal experiments were conducted with the approval of the Institutional Animal Care and Use Committee of the Catholic University of Korea (approval number: CUK-IACUC-2019-026-01). A2780 cell suspension (1 × 10^7^ cells/200 μL in 1 × PBS) was injected subcutaneously into the dorsal scapula area of each mouse. The tumor developed 14 days after implantation to a size of approximately 150 mm^3^. The mice were then divided randomly into vehicle control (5% DMSO), siltuximab (10 mg/kg), and butein (2 or 4 mg/kg) groups. All drugs were dissolved in 0.05% carboxymethylcellulose sodium salt (CMC) (Sigma-Aldrich, St. Louis, MO, USA) and injected intraperitoneally five times a week for three weeks. The tumor size was measured using calipers once every 2 days. The tumor volume was calculated using a simplified equation (length × width^2^ × 0.5). Each tumor was harvested 22 days post-treatment.

### 4.14. Hematoxylin and Eosin (H&E) Staining in Mouse Tumor Tissues

After the experiment was completed, mice were sacrificed and tumor tissues were collected. Collected tumor tissues were fixed in 4% paraformaldehyde for 24 h. Fixed tissues were washed in 1 × PBS and embedded in paraffin. The paraffin block was sectioned at a thickness of 2 μm and sections placed on a slide glass. After hematoxylin and eosin staining, the sections were dehydrated, deparaffinized with mineral oil, and a cover slip was added.

### 4.15. Enzyme-Linked Immunosorbent Assay (ELISA)

An amount of 0.5 μg/mL rhIL-6 protein (Cat.no.570806, BioLegend) was coated onto 96-well ELISA plates at 4 °C overnight, and then the plates were washed with PBST (1 × PBS containing 0.05% Tween-20), followed by the addition of PBSA (1 × PBS containing 1% bovine serum albumin (BSA)) (Bovogen, East Keilor, Australia) for 1 h at room temperature. After blocking, the FDA-approved compounds (20 uM) (Cat.no. L-1300, Selleckchem, Houston, TX, USA) were incubated for 2 h at room temperature. After washing three times with PBST, 0.2 μg/mL recombinant IL-6Ra protein (Cat.no.ILR-H5259, Acro biosystems, Newark, DE, USA) was added to the plates, which were incubated for 1 h. After washing three times with PBST, HRP-conjugated goat anti-human IgG Ab (Cat.no. 10-035-098, Jackson Immno, Hatfield, West Grove, PA, USA) was added to the plates and incubated for 1 h. After washing three times with PBST, TMB solution (Cat.no.11684302001, Roche, Mannheim, Germany) was added to the plates and incubated for 15 min. Then, stop solution (2N HCl) was added to the plates. The absorbance was measured at 450 nm using the Epoch microplate spectrophotometer (BioTek instruments, Winooski, VT, USA).

Concentrations of IL-6, Interleukin-1 beta (IL-1β), and tumor necrosis factor-alpha (TNF-α) in mouse blood serum were measured using an ELISA (mouse IL-6, IL-1β, and TNF-α uncoated ELISA Kit, Invitrogen, MA, USA) kit according to the manufacturer’s protocol. All ELISA analyses were performed in triplicate.

### 4.16. Data Analysis

All data, except that from the cell cycle analysis, were evaluated using GraphPad Prism software (version 7.00; GraphPad Software, Inc., San Diego, CA, USA). Results are presented as mean ± standard deviation (SD). Following two-way ANOVA, Bonferroni’s post hoc tests were performed with GraphPad Prism 7.0.

## 5. Conclusions

Based on our findings, we can conclude that butein from *Butea monosperma* flower isolates inhibits ovarian cancer growth by binding to IL-6 and suppressing its activity, which results in inactivation of STAT3 and nuclear accumulation of FoxO3a and p27^kip1,^ thereby limiting tumor growth. Our work highlights butein as a promising therapeutic agent for ovarian cancer treatment.

## Data Availability

Not applicable.

## Supplementary Materials

The following supporting information can be downloaded at: https://www.mdpi.com/article/10.3390/ijms24076038/s1.

## Abbreviations

IL-6: Interleukin-6; IL-6R: Interleukin-6 receptor; HPLC: high-performance liquid chromatography; HPLCC: high-performance countercurrent chromatography; SPR: surface plasmon resonance; RIPA: radioimmunoprecipitation assay; STAT3: signal transducer and activator of transcription 3; FoxO3a: Forkhead box O3a; NRF: National Research Foundation of Korea.
