# Supplementary material for: Butein Inhibits Cell Growth by Blocking the IL-6/IL-6Rα Interaction in Human Ovarian Cancer and by Regulation of the IL-6/STAT3/FoxO3a Pathway
Source: Int J Mol Sci. 2023 Mar 23;24(7):6038. doi: 10.3390/ijms24076038 (PMC10094418; doi:10.3390/ijms24076038)
Supplement: Supplementary file 1 [file ijms-24-06038-s001.zip › ijms-2154927-supplementary.pdf]

Butein inhibits Cell Growth by Blocking the IL-6/IL-6Rα Interaction in Human Ovarian Cancer and by Regulation of the IL-6/STAT3/FoxO3a Pathway

Supplementary Information

SI 1. Spectroscopic data of compounds 1 – 14.

Liquiritigenin-5′-O-methyl ether (**1**): white amorphous solid; $C_{16}H_{14}O_5$; ESI-Q-TOF- MS: *m/z* 287.0919 $[M+H]^+$; ^{1}H-NMR (500 MHz, methanol-*d*$_4$): d 7.73 (1H, d, *J* = 8.7 Hz, H-5), 7.09 (1H, d, *J* = 2.0 Hz, H-2'), 6.93 (1H, dd, *J* = 8.2, 2.0 Hz, H-6'), 6.82 (1H, d, *J* = 8.1 Hz, H-5'), 6.50 (1H, dd, *J* = 8.8, 2.3 Hz, H-6), 6.37 (1H, d, *J* = 2.2 Hz, H-8), 5.38 (1H, dd, *J* = 13.1, 2.8 Hz, H-2), 3.88 (3H, s, 3'-OCH3), 3.08 (1H, dd, *J* = 16.9, 13.1 Hz, H-3a), 2.70 (1H, dd, *J* = 16.9, 2.9 Hz, H-3b); ^{13}C-NMR (125 MHz, methanol-*d*$_4$): d 193.69 (C-4), 166.96 (C-9), 165.7 (C-7), 149.26 (C-3'), 148.22 (C-4'), 132.15 (C-1'), 130.04 (C-5), 120.68 (C-6'), 116.28 (C-5'), 115.16 (C-10), 111.92 (C-6), 111.42 (C-2'), 103.99 (C-8), 81.44 (C-2), 56.60 (3'-OCH3), 45.25 (C-3).

(+)-Butin (**2**): white amorphous solid; $C_{15}H_{12}O_5$; ESI-Q-TOF-MS: *m/z* 273.0761 $[M+H]^+$

^{1}H-NMR (500 MHz, methanol-*d*$_4$): d 7.72 (1H, d, *J* = 8.7 Hz, H-5), 6.93(1H, d, *J* = 1.8Hz, H-2'), 6.80 (1H, dd, *J* = 7.9, 1.8 Hz, H-6'), 6.78 (1H, d, *J* = 7.9 Hz, H-5'), 6.49 (1H, dd, *J* = 8.7, 2.3 Hz, H-6), 6.35 (1H, d, *J* = 2.2 Hz, H-8), 5.31 (1H, dd, *J* = 12.9, 3.0 Hz, H-2), 3.01 (1H, dd, *J* = 16.9, 12.9 Hz, H-3a), 2.69 (1H, dd, *J* = 16.9, 3.0 Hz, H-3b); ^{13}C-NMR (125 MHz, methanol-*d*$_4$): d 193.54 (C-4), 166.79 (C-7), 165.52 (C-9), 146.81 (C-4'), 146.47 (C-3'), 132.01 (C-1'), 129.84 (C-5), 119.22 (C-6'), 116.21 (C-5'), 114.96 (C-2'), 114.67 (C-10), 111.71 (C-6), 103.8 (C-8), 81.05 (C-2), 45.01 (C-3).

(-)-Isomonospermoside (**3**): white amorphous solid; $C_{21}H_{22}O_{10}$; ESI-Q-TOF-MS: *m/z* 433.114 $[M-H]^-$; ^{1}H-NMR (500 MHz, DMSO-*d*$_6$): d 7.64 (1H, d, *J* = 8.7 Hz, H-5), 7.26 (1H, d, *J* = 2.1 Hz, H-2'), 7.03 (1H, dd, *J* = 8.2, 2.2 Hz, H-6'), 6.85 (1H, d, *J* = 8.2 Hz, H-5'), 6.51 (1H, dd, *J* = 8.7, 2.2 Hz, H-6), 6.35 (1H, d, *J* = 2.4 Hz, H-8), 5.42 (1H, dd, *J* = 12.8, 2.8 Hz, H-2), 4.72 (1H, d, *J* = 7.3 Hz, H-1''), 3.70 (1H, d, *J* = 11.6 Hz, H-6''), 3.48 (1H, dd, *J* = 12.4, 5.7 Hz, H-2''), 3.2-3.71 (1H, o, H-3''), 3.13 (1H, dd, *J* = 16.8, 12.8 Hz, H-3a), 2.65 (1H, dd, *J* = 16.8, 3.0 Hz, H-3b); ^{13}C-NMR (125 MHz, DMSO-*d*$_6$): d 190.15 (C-4), 164.66 (C-7), 163.17 (C-9), 147.01 (C-4'), 145.15 (C-3'), 130.04 (C-1'), 128.42 (C-5), 121.56 (C-6'), 115.76 (C-5'), 115.58 (C-2'), 113.56 (C-10), 110.54 (C-6), 102.63 (C-8), 102.26 (C-1''), 78.89 (C-2), 77.17 (C-5''), 75.99 (C-3''), 73.38 (C-2''), 69.77 (C-4''), 60.66 (C-6''), 43.12 (C-3).

Isocoreopsin (**4**): orange solid; $C_{21}H_{22}O_{10}$; ESI-Q-TOF-MS: *m/z* 435.1290 $[M+H]^+$ ^{1}H-NMR (500 MHz, methanol-*d*$_4$): d 7.80 (1H, d, *J* = 8.8 Hz, H-5), 6.93 (1H, d, *J* = 2.0 Hz, H-2'), 6.81 (1H, dd, *J* = 8.2, 1.9 Hz, H-6'), 6.78 (2H, dd, *J* = 8.4, 2.1 Hz, H-6,5'), 6.71 (1H, d, *J* = 2.3 Hz, H-8), 5.36 (1H, dd, *J* = 13.0, 3.2 Hz, H-2), 5.01 (1H, d, *J* = 7.6 Hz, H-1''), 3.68 (1H, dd, *J* = 12.2, 4.0 Hz, H-6''b), 3.52 - 3.43 (5H, m, H-2'',3'',4'',5'',6''a), 3.07 (1H, dd, *J* = 16.9, 12.9 Hz, H-3a), 2.74 (1H, dd, *J* = 16.9, 2.9 Hz, H-3b); ^{13}C-NMR (125 MHz, methanol-*d*$_4$): d 193.57 (C-4), 165.59 (C-7), 165.06 (C-9), 146.91 (C-4'), 146.52 (C-3'), 131.8 (C-1'), 129.46 (C-5), 119.32 (C-6'), 116.99 (C-10), 116.2 (C-5'), 114.76 (C-2'), 112.18 (C-6), 105.23 (C-8), 101.42 (C-1''), 81.34 (C-2), 78.28 (C-5''), 77.83 (C-3''), 74.70 (C-2''), 71.20 (C-4''), 62.37 (C-6''), 44.92 (C-3).

Liquiritigenin-7-O-B-D-glucopyranoside (**5**): orange solid; $C_{21}H_{22}O_9$; ESI-Q-TOF-MS: *m/z* 417.122 $[M-H]^-$; ^{1}H-NMR (500 MHz, methanol-*d*$_4$): d 7.81 (1H, d, *J* = 8.8 Hz, H-5), 7.33 (2H, d, *J* = 8.6 Hz, H-2',6'), 6.82 (2H, d, *J* = 8.6 Hz, H-3',5'), 6.78 (1H, dd, *J* = 8.8, 2.3 Hz, H-6), 6.71 (1H, d, *J* = 2.4 Hz, H-8), 5.42 (1H, dd, *J* = 13.1, 2.4 Hz, H-2), 5.01 (1H, d, *J* = 7.7 Hz, H-1''), 3.88 (1H, dd, *J* = 12.2, 2.1 Hz, H-6''a), 3.68 (1H, dd, *J* = 12.1, 4.4 Hz, H-6''b), 3.34-3.51 (4H, m, H-2'',3'',4'',5''), 3.11 (1H, dd, *J* = 16.9, 13.2 Hz, H-3a), 2.73 (1H, dd,

J = 16.9, 2.9 Hz, H-3b); ^{13}C-NMR (125 MHz, methanol-d_4): d 193.6 (C-4), 165.58 (C-7), 165.11 (C-9), 159.06 (C-4'), 131.07 (C-1'), 129.43 (C-5), 129.10 (C-2'), 129.1 (C-6'), 116.95 (C-10), 116.29 (C-3'), 116.29 (C-5'), 112.2 (C-6), 105.21 (C-8), 101.41 (C-1''), 81.23 (C-2), 78.26 (C-5''), 77.81 (C-3''), 74.69 (C-2''), 71.18 (C-4''), 62.35 (C-6''), 45.00 (C-3).

(-)-Butrin (**6**): white solid; $C_{27}H_{32}O_{15}$; ESI-Q-TOF-MS: *m/z* 595.166 $[M-H]^-$; ^{1}H-NMR (500 MHz, DMSO-d_6): d 7.72 (1H, d, J = 8.8 Hz, H-5), 7.29 (1H, d, J = 2.0 Hz, H-2'), 7.03 (1H, dd, J = 8.4, 2.0 Hz, H-6'), 6.84 (1H, d, J = 8.2 Hz, H-5'), 6.71 (1H, dd, J = 8.7, 2.3 Hz, H-6), 6.67 (1H, d, J = 2.4 Hz, H-8), 5.46 (1H, dd, J = 13.1, 2.7Hz, H-2), 4.99 (1H, d, J = 7.4 Hz, H-1''), 4.72 (1H, d, J = 7.3Hz, H-1'''), 3.69 (2H, o, H-6'', 6'''a), 3.46 (1H, o, H-6'''b), 3.38 (1H, o, H-5''), 3.35 (1H, o, H-3''), 3.31 (1H, o, H-2'''), 3.28 (1H, o, H-5'''), 3.26 (1H, o, H-3'''), 3.24 (1H, o, H-2''), 3.21 (1H, o, H-3a), 3.17 (2H, o, H-4'',4'''), 2.68 (1H, dd, J = 17.0, 3.5 Hz, H-3b); ^{13}C-NMR (125 MHz, DMSO-d_6): d 190.58 (C-4), 163.42 (C-7), 162.98 (C-9), 147.21 (C-4'), 145.15 (C-3'), 129.67 (C-1'), 127.95 (C-5), 121.79 (C-6'), 115.82 (C-2'), 115.33 (C-5'), 115.3 (C-10), 110.94 (C-6), 103.55 (C-8), 102.32 (C-1'''), 99.71 (C-1''), 79.21 (C-2), 77.22 (C-5''), 77.02 (C-3''), 76.4 (C-5'''), 75.99 (C-3'''), 73.38 (C-2'''), 73.11 (C-2''), 69.91 (C-4'''), 69.45 (C-4''), 60.7 (C-6'''), 60.53 (C-6''), 43.06 (C-3).

3′,4′,7-Trihydroxyflavone-7-O-glucoside (**7**): orange solid; $C_{21}H_{20}O_{10}$; ESI-Q-TOF-MS: *m/z* 431.099 $[M-H]^-$; ^{1}H-NMR (500 MHz, methanol-d_4): d 7.68 (1H, d, J = 8.6 Hz, H-5), 7.51 (1H, d, J = 2.1 Hz, H-2'), 7.26 (1H, dd, J = 8.3, 2.1 Hz, H-6'), 7.08 (1H, d, J = 2.0 Hz, H-8), 6.96 (1H, dd, J = 8.6, 2.0 Hz, H-6), 6.84 (1H, d, J = 8.3 Hz, H-5'), 6.74 (1H, s, H-3), 5.12 (1H, d, J = 7.7 Hz, H-1''), 3.95 (1H, m, H-6''a), 3.73 (1H, dd, J = 12.1, 5.8 Hz, H-6''b), 3.60–3.48 (3H, m, H-2'',3'',5''), 3.47–3.37 (1H, m, H-4''); ^{13}C-NMR (125 MHz, methanol-d_4): d 184.59 (C-4), 169.08 (C-9), 166.64 (C-7), 149.67 (C-4'), 147.44 (C-2), 146.79 (C-3'), 126.6 (C-1'), 126.4 (C-6'), 125.35 (C-5), 119.1 (C-2'), 117.17 (C-10), 116.68 (C-5'), 115.53 (C-3), 114.79 (C-6), 101.7 (C-1''), 100.7 (C-8), 78.42 (C-5''), 77.81 (C-3''), 74.69 (C-2''), 71.2 (C-4''), 62.44 (C-6'').

Isoliquilitigenin (**8**): yellowish solid; $C_{15}H_{12}O_4$; ESI-Q-TOF-MS: *m/z* 257.0814 $[M+H]^+$; ^{1}H-NMR (500 MHz, methanol-d_4): d 7.97 (1H, d, J = 9 Hz, H-2'), 7.78 (1H, d, J = 15.4 Hz, H-7), 7.61 (2H, d, J = 8.9 Hz, H-2,6), 7.6 (1H, d, J = 15.4 Hz, H-8), 6.85 (2H, d, J = 8.8 Hz, H-3,5), 6.41 (1H, dd, J = 8.9, 2.4 Hz, H-3'), 6.29 (1H, d, J = 2.3 Hz, H-5'); ^{13}C-NMR (125 MHz, methanol-d_4): d 193.51 (C-9), 167.51 (C-2'), 166.35 (C-4'), 161.56 (C-4), 145.65 (C-7), 133.37 (C-6'), 131.84 (C-2,6), 127.83 (C-1), 118.29 (C-8), 116.9 (C-3,5), 114.70 (C-1'), 109.12 (C-5'), 103.79 (C-3').

Butein (**9**): orange solid; $C_{15}H_{12}O_5$; ESI-Q-TOF-MS: *m/z* 271.0606 $[M-H]^{-;}$ ^{1}H-NMR (500 MHz, methanol-d_4): d 7.94 (1H, d, J = 8.9 Hz, H-6'), 7.72 (1H, d, J = 15.2 Hz, H-7), 7.54 (1H, d, J = 15.4 Hz, H-8), 7.18 (1H, d, J = 2.0 Hz, H-2), 7.11 (1H, dd, J = 8.3, 2.1 Hz, H-6), 6.82 (1H, d, J = 8.2 Hz, H-5), 6.42 (1H, dd, J = 8.9, 2.4 Hz, H-5'), 6.29 (1H, d, J = 2.4 Hz, H-3'); ^{13}C-NMR (125 MHz, methanol-d_4): d 193.48 (C-9), 167.52 (C-2'), 166.36 (C-4'), 149.95 (C-3), 146.86 (C-4), 146.1 (C-7), 133.31 (C-6'), 128.42 (C-1), 123.63 (C-6), 118.27 (C-8), 116.61 (C-5), 115.81 (C-2), 114.71 (C-1'), 109.14 (C-5'), 103.82 (C-3').

Homobutein (**10**): yellowish solid; $C_{16}H_{13}O_5$; ESI-Q-TOF-MS: *m/z* 285.0770 $[M-H]^-$; ^{1}H- NMR (500 MHz, methanol-d_4): d 8.03 (1H, d, J = 8.7 Hz, H-6'), 7.78 (1H, d, J = 15.1 Hz, H-α), 7.65 (1H, d, J = 15.1 Hz, H-β), 7.37 (1H, brs, H-2), 7.22 (1H, d, J = 8.0 Hz, H-6), 6.84 (1H, d, J = 7.8 Hz, H-5), 6.41 (1H, dd, J = 8.7, 2.3 Hz, H-5'), 6.29 (1H, d, J = 2.1 Hz, H-3'), 3.94 (3H, s, 3-OCH_3); ^{13}C-NMR (125 MHz, methanol-d_4 d 193.5 (C=O), 167.53 (C-2'), 166.36 (C-4'), 150.95 (C-3), 149.44 (C-4), 145.99 (C-α), 133.5 (C-6'), 128.42 (C-1), 125.01 (C-6), 118.62 (C-β), 116.52 (C-5), 114.74 (C-1'), 112.15 (C-2), 109.09 (C-5'), 103.77 (C-3'), 56.51 (3-OCH3).

Monospermoside (**11**): yellowish solid; $C_{21}H_{22}O_{10}$; ESI-Q-TOF-MS: *m/z* 435.1300 $[M+H]^+$; ^{1}H-NMR (500 MHz, methanol-d_4): d 8.00 (1H, d, J = 8.8 Hz, H-6'), 7.76(1H, d, J = 15.4Hz, H-β), 7.71 (1H, d, J = 2.0 Hz, H-2), 7.62 (1H, d, J = 15.4Hz, H-α), 7.30 (1H, dd, J = 8.3, 2.0 Hz, H-6), 6.90 (1H, d, J = 8.2 Hz, H-5), 6.42 (1H, dd, J = 8.9, 2.4 Hz, H-5'), 6.28 (1H, d, J = 2.4 Hz, H-3'), 4.88 (1H, d, J = 7.2 Hz, H-1''), 3.99 (1H, dd, J

= 12.0, 2.2 Hz, H-6''b), 3.73 (1H, dd, *J* = 12.0, 6.6 Hz, H-6''a), 3.58 – 3.46 (3H, m, H-2'',3'',5''), 3.41 – 3.36 (1H, m, H-4''); ^{13}C-NMR (125 MHz, methanol-d_4): d 193.44 (C=O), 167.52 (C-2'), 166.39 (C-4'), 151.3 (C-4), 147.48 (C-3), 145.41 (C-β), 133.62 (C-6'), 128.63 (C-1), 127.13 (C-6), 119.16 (C-α), 118.01 (C-2), 117.43 (C-5), 114.69 (C-1'), 109.18 (C-5'), 104.25 (C-1''), 103.74 (C-3'), 78.66 (C-5''), 77.62 (C-3''), 74.92 (C-2''), 71.66 (C-4''), 62.7 (C-6'').

Isoliquiritin (**12**): white amorphous solid; $C_{21}H_{22}O_9$; ESI-Q-TOF-MS: *m/z* 419.1350 $[M+H]^+$ ^{1}H-NMR (500 MHz, methanol-d_4): d 8.00 (1H, d, *J* = 8.7 Hz, H-6), 7.82 (1H, d, *J* = 15.3 Hz, H-β), 7.73 (2H, d, *J* = 8.5 Hz, H-2′, H-6′), 7.70 (1H, d, *J* = 15.3 Hz, H-α), 7.16 (2H, d, *J* = 8.5 Hz, H-3′, H-5′), 6.43 (1H, dd, *J* = 8.7, 2.2 Hz, H-5), 6.30 (1H, d, *J* = 2.2 Hz, H-3), 5.00 (1H, dd, *J* = 5.3, 2.3 Hz, H-1''), 3.91 (1H, dd, *J* = 12.1, 2.1 Hz, H-6''b), 3.71 (1H, dd, *J* = 12.1, 5.6 Hz, H-6''a), 3.49 (3H, qd, *J* = 6.9, 3.2 Hz, H-2'',3'',5''), 3.40 (1H, ddd, *J* = 9.4, 5.9, 2.9 Hz, H-4''); ^{13}C-NMR (125 MHz, methanol-d_4): d 193.38 (C=O), 167.62 (C-2), 166.54 (C-4), 161.08 (C-4'), 144.79 (C-β), 133.51 (C-6), 131.41 (C-2',6'), 130.55 (C-1'), 120.05 (C-α), 118 (C-3', 5'), 114.68 (C-1), 109.21 (C-5), 103.79 (C-3), 101.82 (C-1''), 78.28 (C-5''), 77.96 (C-3''), 74.84 (C-2''), 71.3 (C-4''), 62.47 (C-6'').

Coreopsin (**13**): orangish powder; $C_{21}H_{22}O_{10}$; ESI-Q-TOF-MS: *m/z* 435.1290 $[M+H]^{+;}$ ^{1}H-NMR (500 MHz, methanol-d_4): d 8.04 (1H, d, *J* = 9.1 Hz, H-6'), 7.76(1H, d, *J* = 15.2Hz, H-β), 7.57 (1H, d, *J* = 15.3 Hz, H-a), 7.20 (1H, d, *J* = 2.0 Hz, H-2), 7.13 (1H, dd, *J* = 8.2, 2.0 Hz, H-6), 6.82 (1H, d, *J* = 8.1 Hz, H-5), 6.69 (1H, dd, *J* = 8.9, 2.4 Hz, H-5'), 6.62 (1H, d, *J* = 2.5 Hz, H-3'), 5.04 (1H, d, *J* = 7.1 Hz, H-1''), 3.91 (1H, dd, *J* = 12.1, 2.2 Hz, H-6''a), 3.72 (1H, dd, *J* = 12.2, 5.4 Hz, H-6''b), 3.53 – 3.47 (3H, m, H- 2'',3'',5''), 3.45 - 3.37 (1H, m, H-4''); ^{13}C-NMR (125 MHz, methanol-d_4): d 192.5 (C-β'), 165.46 (C-2'), 163.67 (C-4'), 148.76 (C-4), 145.48 (C-3), 145.46 (C-β), 131.50 (C-6'), 126.89 (C-1), 122.47 (C-6), 116.66 (C-α), 115.22 (C-1'), 115.19 (C-5), 114.49 (C-2), 107.91 (C-5'), 103.75 (C-3'), 99.91 (C-1''), 76.9 (C-5''), 76.47 (C-3''), 73.33 (C-2''), 69.77 (C-4''), 60.96 (C-6'').

Isobutrin (**14**): yellowish solid; $C_{27}H_{32}O_{15}$; ESI-Q-TOF-MS: *m/z* 595.1671 $[M-H]^-$; ^{1}H-NMR (500 MHz, methanol-d_4): d 8.08 (1H, d, *J* = 9.1 Hz, H-6'), 7.79 (1H, d, *J* = 15.3 Hz, H-α), 7.71 (1H, d, *J* = 2.1 Hz, H-2), 7.63(1H, d, *J* = 15.3 Hz, H-β), 7.31 (1H, dd, *J* = 8.3, 2.0 Hz, H-6), 6.89 (1H, d, *J* = 8.3 Hz, H-5), 6.68 (1H, dd, *J* = 9.0, 2.5 Hz, H-5'), 6.60 (1H, d, *J* = 2.4 Hz, H-3'), 5.04 (1H, d, *J* = 7.4 Hz, H-1''), 4.88 (1H, d, *J* = 7.6 Hz, H-1'''), 3.99 (1H, dd, *J* = 12.0, 2.2 Hz, H-6'''a), 3.91 (1H, dd, *J* = 12.1, 2.2 Hz, H-6''a), 3.72 (2H, o, H-6''b), 3.72 (2H, o, H-6'''b), 3.60 – 3.44 (6H, o, H-2'',3'',5'',2''',3''',5'''), 3.46–3.35 (2H, o, H-4'',4'''); ^{13}C-NMR (125 MHz, methanol-d_4): d 193.87 (C=O), 166.85 (C-2'), 165.08 (C-4'), 151.49 (C-4), 147.22 (C-3), 146.19 (C-α), 133.19 (C-6'), 128.46 (C-1), 127.34 (C-6), 118.87 (C-β), 118 (C-2), 117.46 (C-5), 116.54 (C-1'), 109.37 (C-5'), 105.06 (C-3'), 104.16 (C-1'''), 101.26 (C-1''), 78.67 (C-5'''), 78.25 (C-5''), 77.82 (C-3''), 77.59 (C-3'''), 74.91 (C-2'''), 74.7 (C-2''), 71.66 (C-4'''), 71.12 (C-4''), 62.72 (C-6'''), 62.32 (C-6'').

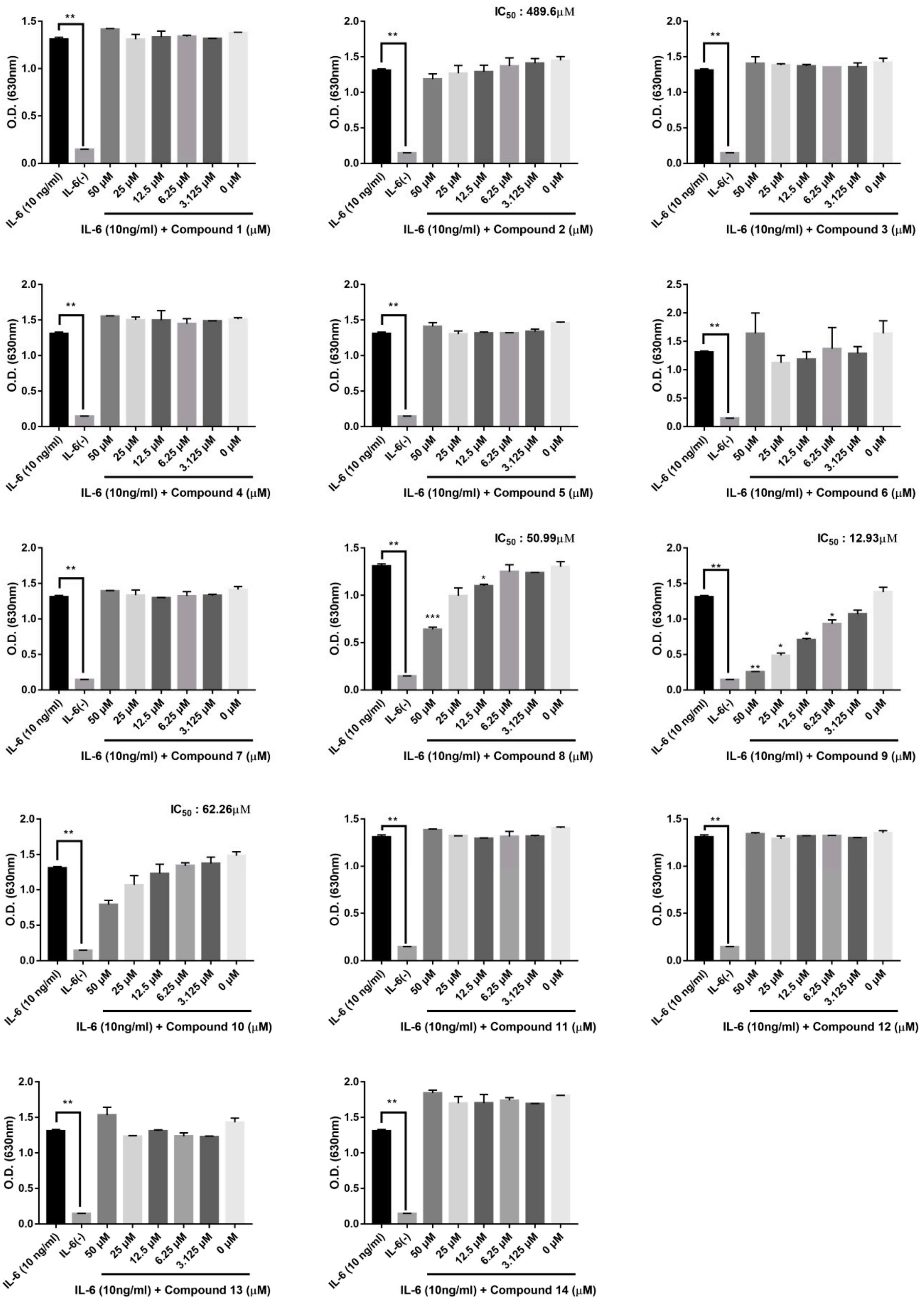

O.D. (630nm)
IL-6 (10 ng/ml)
IL-6(-)
50 μM
25 μM
12.5 μM
6.25 μM
3.125 μM
0 μM
IL-6 (10ng/ml) + Compound 1 (μM)
IC50 : 489.6μM
IL-6 (10ng/ml) + Compound 2 (μM)
IL-6 (10ng/ml) + Compound 3 (μM)
IL-6 (10ng/ml) + Compound 4 (μM)
IL-6 (10ng/ml) + Compound 5 (μM)
IL-6 (10ng/ml) + Compound 6 (μM)
IL-6 (10ng/ml) + Compound 7 (μM)
IC50 : 50.99μM
IL-6 (10ng/ml) + Compound 8 (μM)
IC50 : 12.93μM
IL-6 (10ng/ml) + Compound 9 (μM)
IC50 : 62.26μM
IL-6 (10ng/ml) + Compound 10 (μM)
IL-6 (10ng/ml) + Compound 11 (μM)
IL-6 (10ng/ml) + Compound 12 (μM)
IL-6 (10ng/ml) + Compound 13 (μM)
IL-6 (10ng/ml) + Compound 14 (μM)

Figure S1. Screening of IL-6 signaling inhibitory effects on 14 *B. monosperma* isolates using HEK-Blue™ IL-6 cell line. HEK-Blue™ IL-6 cells were treated with various concentration of isolates (0, 3.125, 6.25, 12.5, 25, or 50 μM) for 24 h. The activation of IL-6 was measured by a SEAP activity assay after the treatment of HEK-Blue™ IL-6 cells with the different indicated concentrations of isolates for 1 h in the presence or absence of IL-6 for 24 h. Data are representative of results obtained from three independent HEK-Blue™ assay, and three replicates were analyzed in each assay (##P < 0.01 vs IL-6 (10 ng/ml), *P < 0.05, **P < 0.01, ***P < 0.001 vs IL-6 (-)).

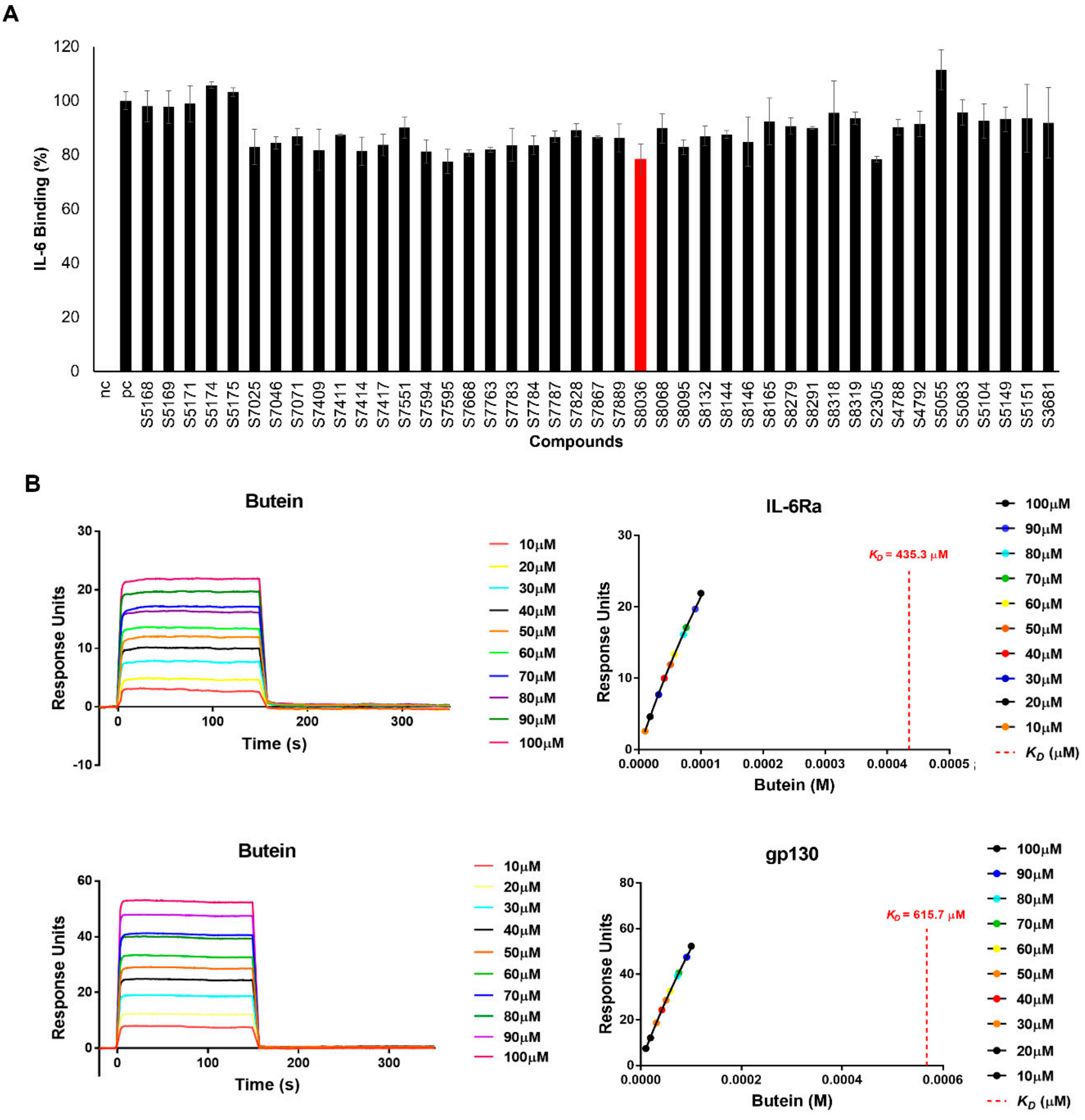

Figure S2. Characterization of butein. (A) Butein interfered with the interaction between IL-6 and IL-6Rα. (B) For SPR analysis, butein binding to IL-6Rα and gp130 were immobilized on a CM5 sensor chip. Butein was injected into the flow cells. T-200 BIAevaluation software was used to subtract the references and determine the steady-state K_D. IL-6Rα, interleukin 6 receptor alpha.

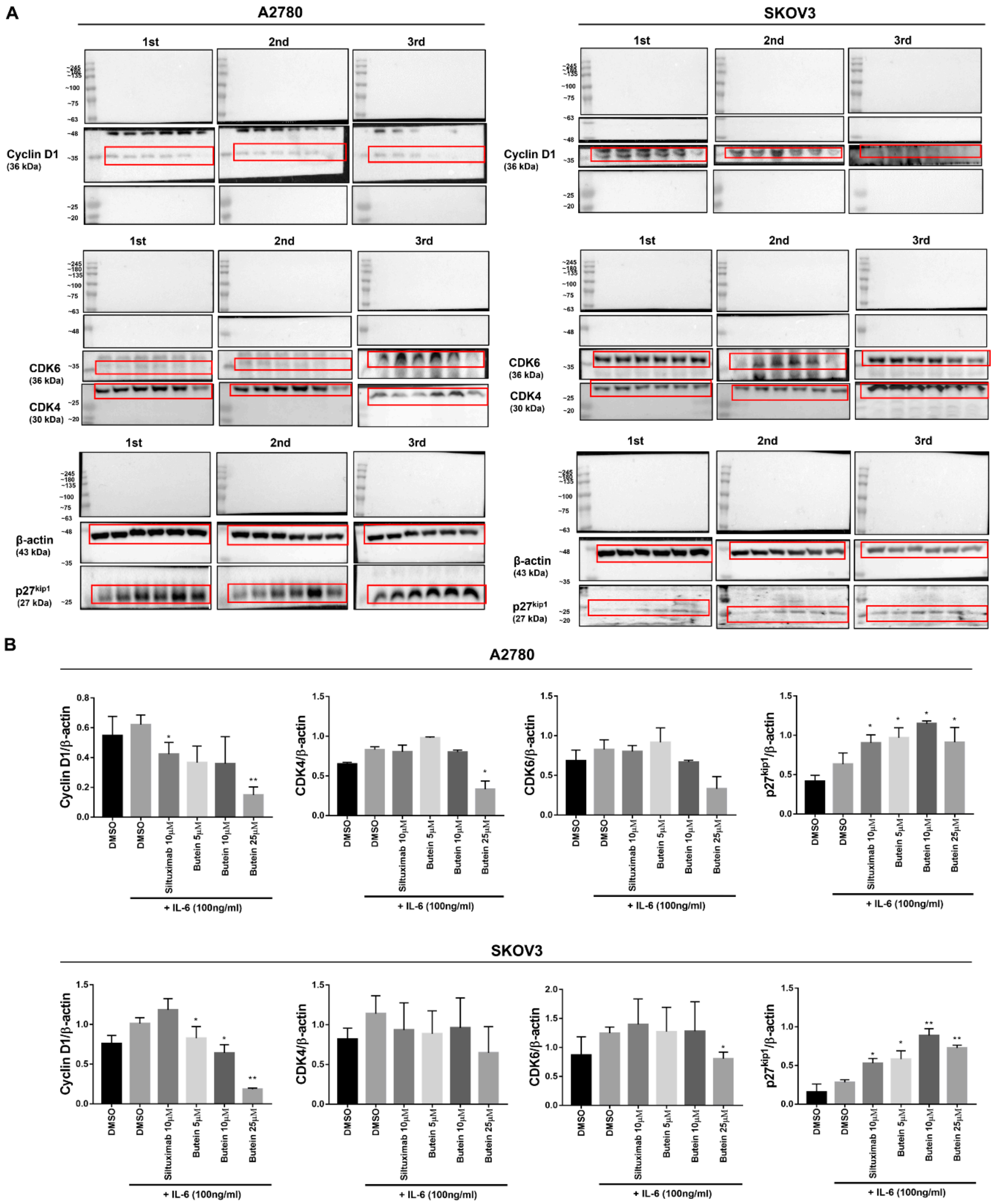

A
A2780
SKOV3
1st
2nd
3rd
Cyclin D1
(36 kDa)
CDK6
(36 kDa)
CDK4
(30 kDa)
β-actin
(43 kDa)
p27kip1
(27 kDa)
B
Cyclin D1/β-actin
CDK4/β-actin
CDK6/β-actin
p27kip1/β-actin
DMSO
Siltuximab 10μM
Butein 5μM
Butein 10μM
Butein 25μM
+ IL-6 (100ng/ml)

C

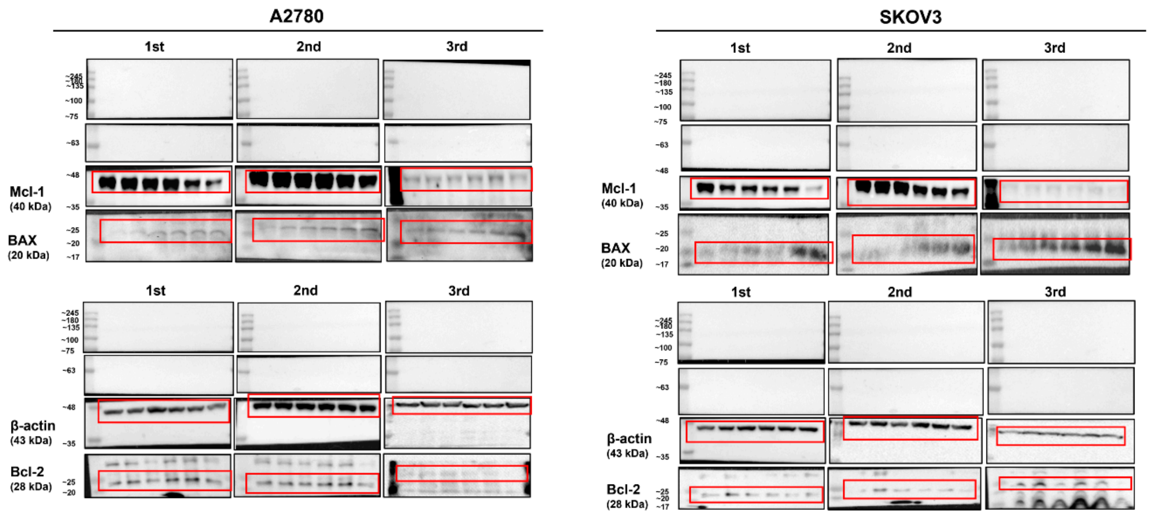

D

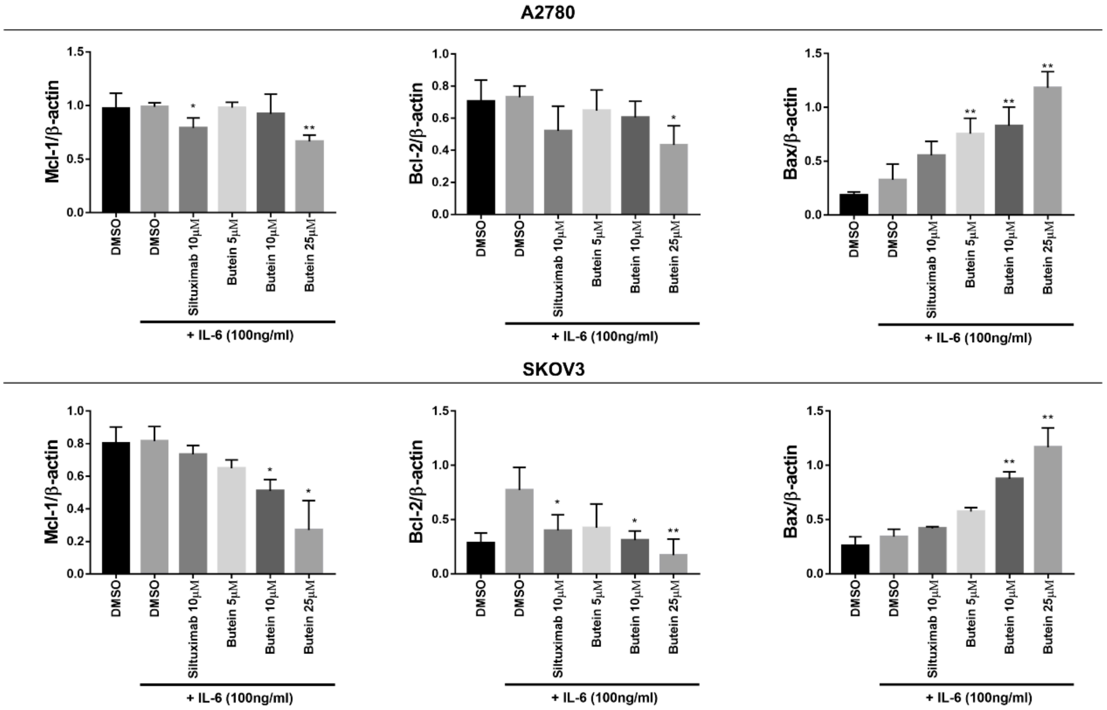

Figure S3. The whole western blot for A2780 and SKOV3 cells in Fig. 4. (A) The whole western blot membrane for Fig. 4C. (B) Levels of cyclin D1, CDK4, CDK6, and p27[kip1] analyzed by western blotting in A2780 and SKOV3 cells (*P < 0.05 and **P < 0.01 identified by two-way ANOVA vs DMSO + IL-6). (C) The whole western blot membrane for Fig. 4D. (D) Levels of Mcl 1, Bcl 2, and Bax analyzed by western blotting in A2780 and SKOV3 cells. Data are presented as mean ± SD from three independent experiments (*P < 0.05, **P < 0.01, and ***P < 0.001 identified by two-way ANOVA vs DMSO + IL-6). CDK4, cyclin-dependent kinase 4; CDK6, cyclin-dependent kinase 6; Bax, Bcl-2-associated X protein; IL, interleukin.

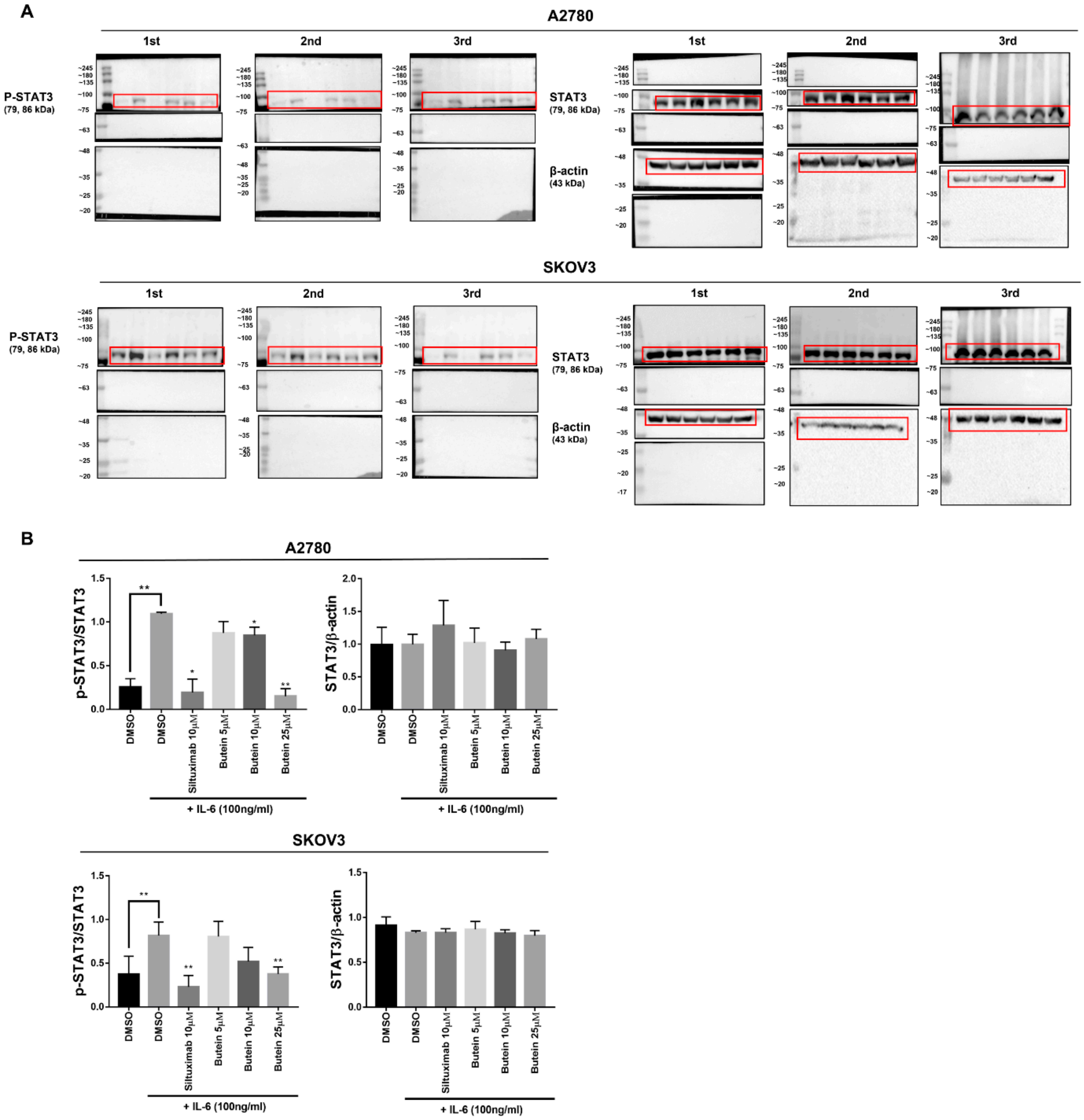

Figure S4. The whole western blot for A2780 and SKOV3 cells in Fig.5A. (A) The whole western blot membrane for Fig.5B. (B) Levels of phosphorylated STAT3 and STAT3 as analyzed by western blotting in A2780 and SKOV3 cells. Data are presented as mean ± SD from three independent experiments (*P < 0.05 and **P < 0.01 identified by two-way ANOVA vs DMSO + IL-6). STAT3, signal transducer and activator of transcription 3; IL, interleukin.

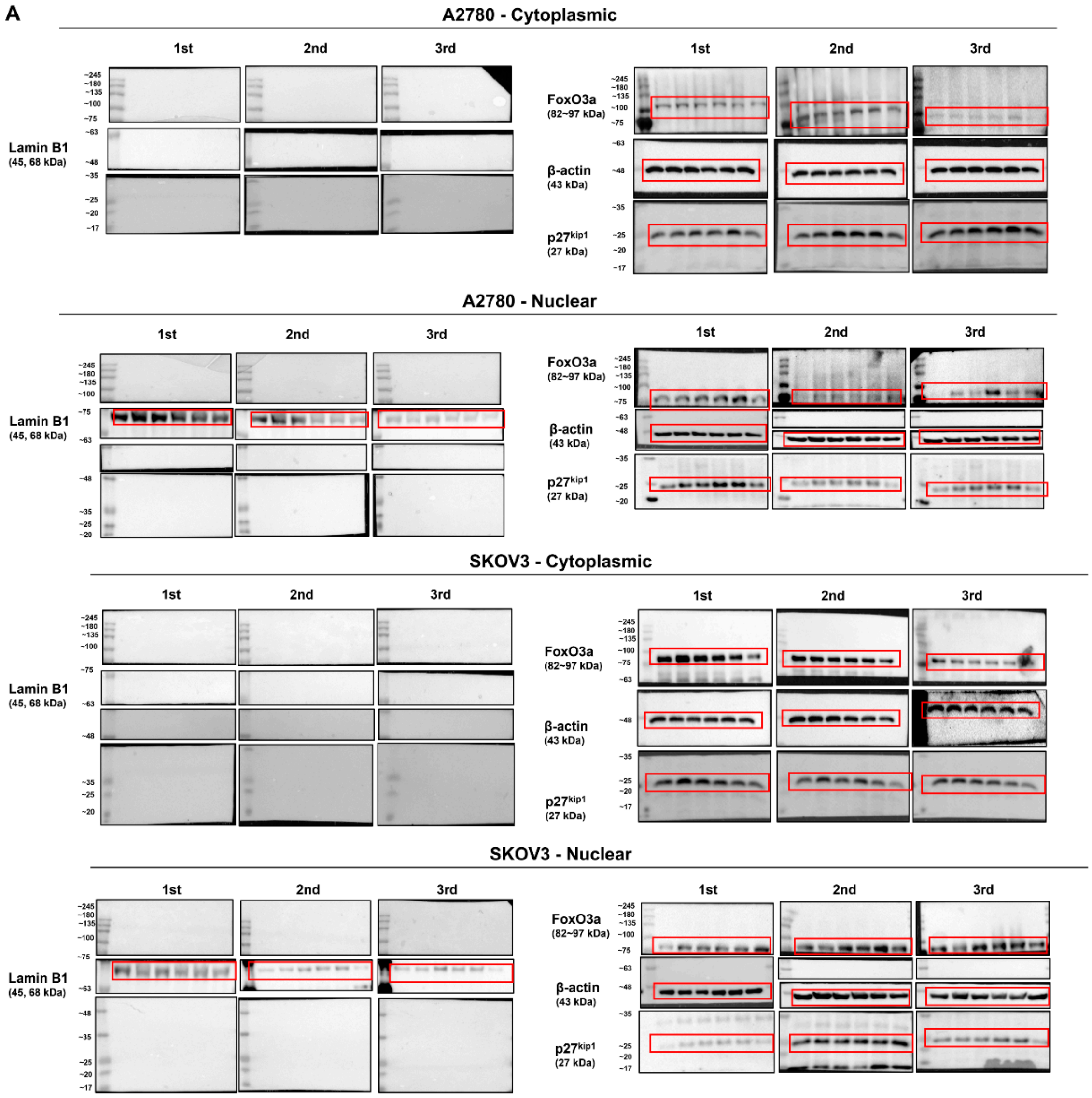
A
A2780 - Cytoplasmic
1st
2nd
3rd
Lamin B1
(45, 68 kDa)
FoxO3a
(82~97 kDa)
β-actin
(43 kDa)
p27kip1
(27 kDa)
A2780 - Nuclear
SKOV3 - Cytoplasmic
SKOV3 - Nuclear

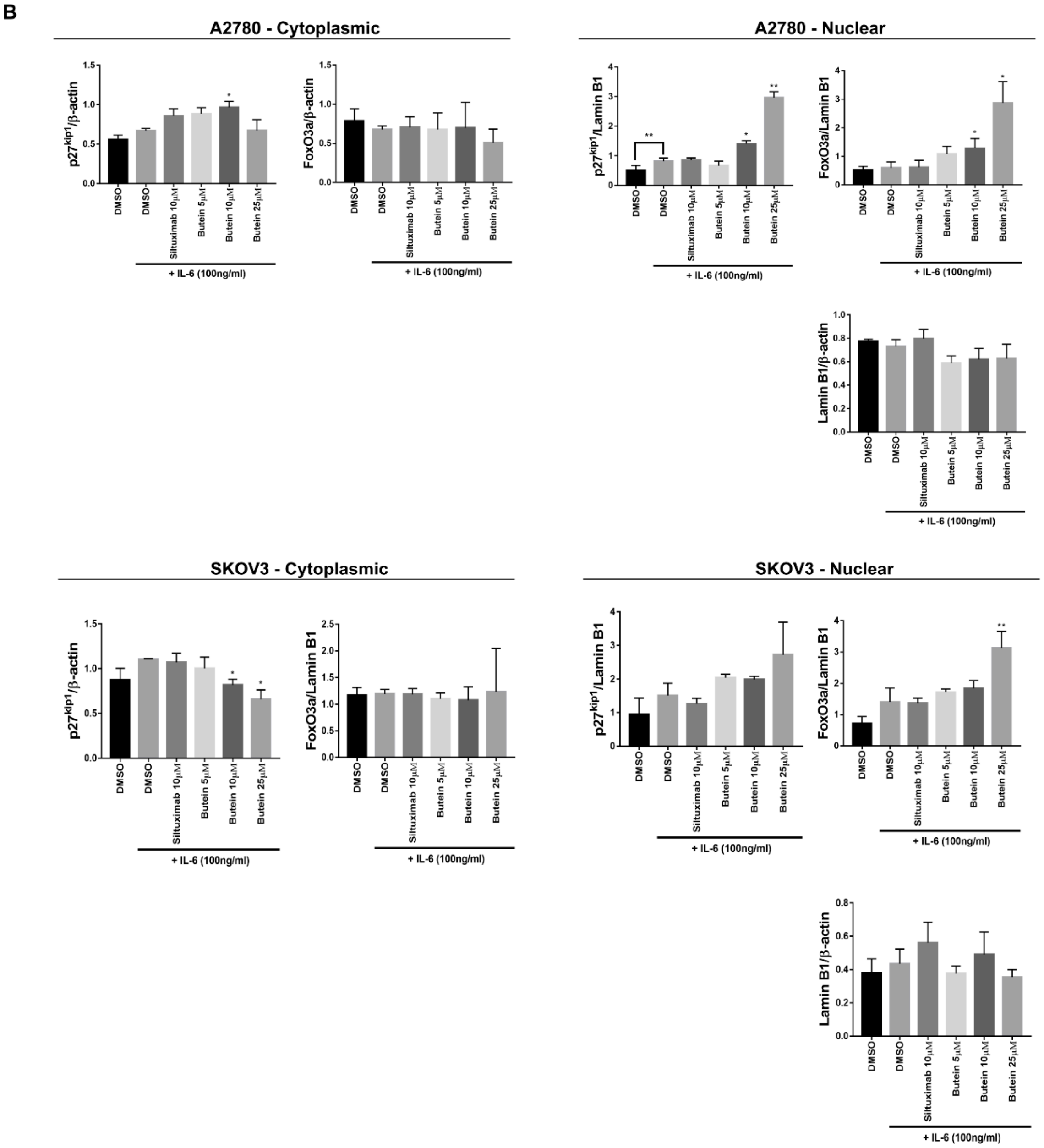

Figure S5. The whole western blot for A2780 and SKOV3 cells in Fig.5B. (A) The whole western blot membrane from the analysis of cytosolic and nuclear extracts of A2780 and SKOV3 cells. Levels of FoxO3a, and p27^{kip1} as analyzed by western blotting analysis of the nuclear extracts from A2780 and SKOV3 cells. Data are presented as mean ± SD from three independent experiments (*P < 0.05 and **P < 0.01 identified by two-way ANOVA vs DMSO + IL-6). FoxO3a, forkhead box 3a; IL, interleukin.

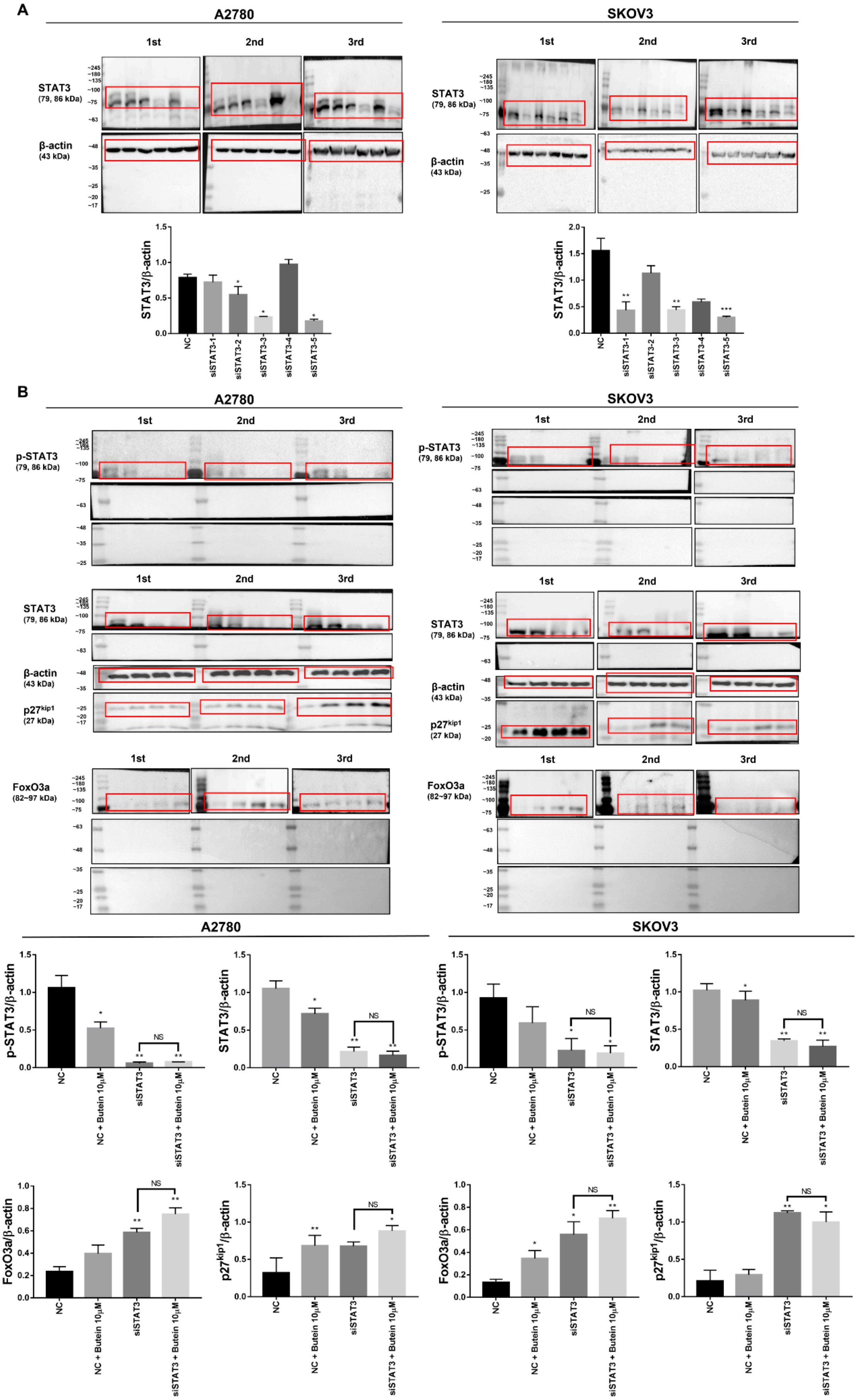
A
A2780
SKOV3
1st
2nd
3rd
STAT3 (79, 86 kDa)
β-actin (43 kDa)
STAT3/β-actin
NC
siSTAT3-1
siSTAT3-2
siSTAT3-3
siSTAT3-4
siSTAT3-5
B
p-STAT3 (79, 86 kDa)
p27kip1 (27 kDa)
FoxO3a (82~97 kDa)
p-STAT3/β-actin
FoxO3a/β-actin
p27kip1/β-actin
NC + Butein 10μM
siSTAT3
siSTAT3 + Butein 10μM
NS

Figure S6. The whole western blot for A2780 and SKOV3 cells in Fig.6. (A) The whole western blot membrane for Fig. 6A. Levels of STAT3 as analyzed by western blotting in A2780 and SKOV3 cells (*$P < 0.05$, **$P < 0.01$ and ***$P < 0.001$ identified by two-way ANOVA vs NC). (B) The whole western blot membrane by western blotting in A2780 and SKOV3 cells in Fig. 6E. (C) Levels of phosphorylated STAT3, STAT3, FoxO3a, and $p27^{kip1}$ as analyzed by western blotting in A2780 and SKOV3 cells. Data are presented as mean ± SD from three independent experiments (*$P < 0.05$ and **$P < 0.01$ identified by two-way ANOVA vs NC; NS, not significant). STAT3, signal transducer and activator of transcription 3; FoxO3a, forkhead box 3a; IL, interleukin.

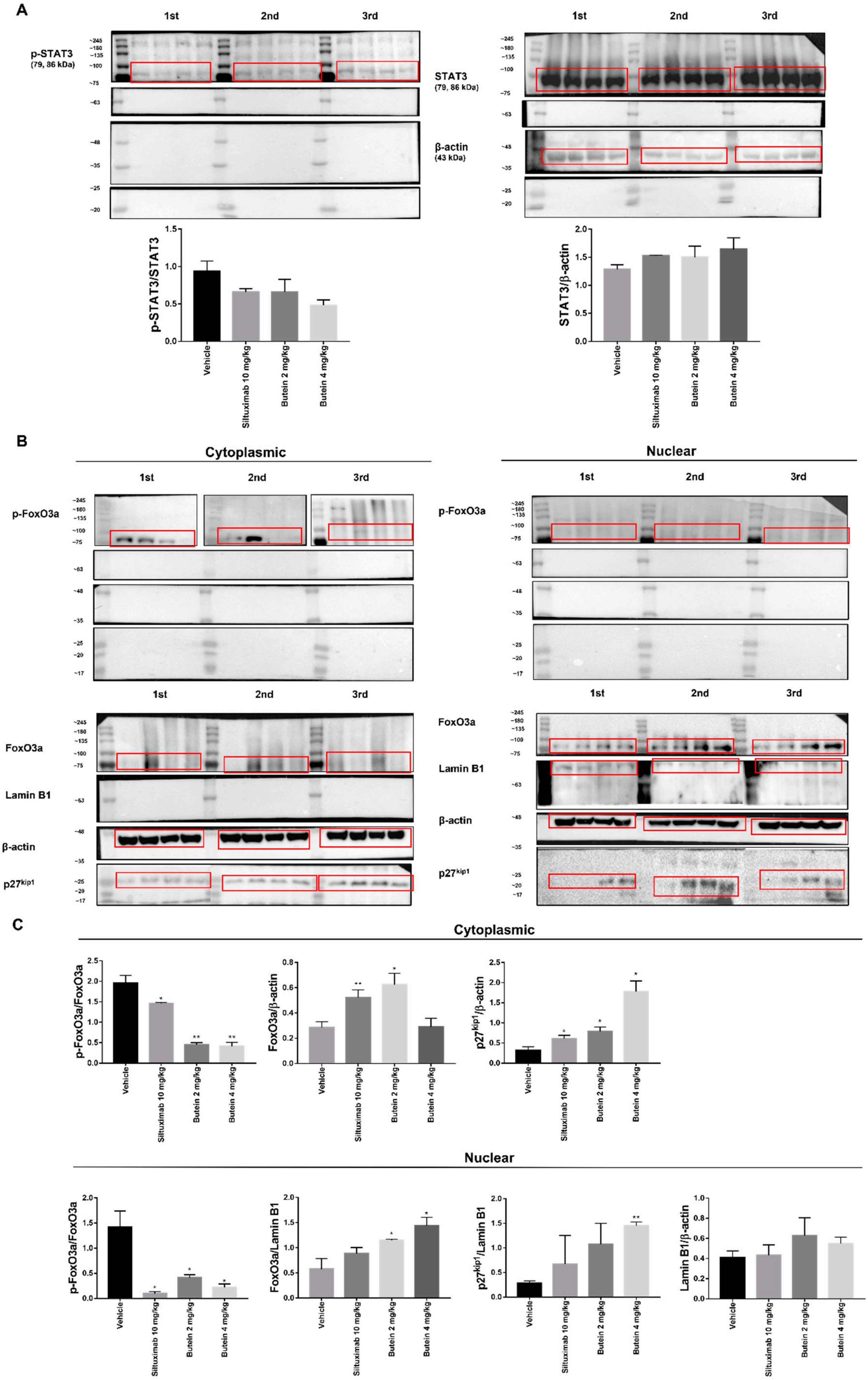
A
1st
2nd
3rd
p-STAT3
(79, 86 kDa)
STAT3
(79, 86 kDa)
β-actin
(43 kDa)
p-STAT3/STAT3
STAT3/β-actin
Vehicle
Siltuximab 10 mg/kg
Butein 2 mg/kg
Butein 4 mg/kg
B
Cytoplasmic
Nuclear
p-FoxO3a
FoxO3a
Lamin B1
β-actin
$p27^{kip1}$
C
Cytoplasmic
p-FoxO3a/FoxO3a
FoxO3a/β-actin
$p27^{kip1}$/β-actin
Nuclear
p-FoxO3a/FoxO3a
FoxO3a/Lamin B1
$p27^{kip1}$/Lamin B1
Lamin B1/β-actin

Figure S7. The whole western blot for Fig.7. (A) The whole western blot membrane for Fig. 7G. Levels of p-STAT3 and STAT3 as analyzed by western blotting analysis of the cytoplasmic extracts from tumor tissues. (B) Levels of FoxO3a, and $p27^{kip1}$ as analyzed by western blotting analysis of the nuclear fractions from tumor tissues (Fig. 7H). Data are presented as mean ± SD from three independent experiments (*P < 0.05 and **P < 0.01 identified by two-way ANOVA vs Vehicle). STAT3, signal transducer and activator of transcription 3; FoxO3a, forkhead box 3a; IL, interleukin.

Table S1. siRNA primer sequences used in the present study.

No	siRNA name		Duplex sequence
1	STAT3-1	Sense	5′ ACAGGAUGGCCCAAUGGAAUU 3′
		Antisense	5′ UUCCAUUGGGCCAUCCUGUUU 3′
2	STAT3-2	Sense	5′ CCAACAAUCCCAAGAAUGUUU 3′
		Antisense	5′ ACAUUCUUGGGAUUGUUGGUU 3′
3	STAT3-3	Sense	5′ AACAUUCUGGGCACAAACAUU 3′
		Antisense	5′ UGUUUGUGCCCAGAAUGUUUU 3′
4	STAT3-4	Sense	5′ GCAAGAUCUGAAUGGAAACUU 3′
		Antisense	5′ GUUUCCAUUCAGAUCUUGCUU 3′
5	Negative Control	Sense	5′ CCUCGUGCCGUUCCAUCAGGUAGUU 3′
		Antisense	5′ CUACCUGAUGGAACGGCACGAGGUU 3′

STAT3, signal transducer and activator of transcription 3.
